# Comparative transcriptomic analysis provides key genetic resources in clove basil (*Ocimum gratissimum*) under cadmium stress

**DOI:** 10.3389/fgene.2023.1224140

**Published:** 2023-07-27

**Authors:** Bin Wang, Yukun Wang, Xiao Yuan, Yuanyuan Jiang, Yunna Zhu, Xinmiao Kang, Jinming He, Yanhui Xiao

**Affiliations:** ^1^ Guangdong Provincial Key Laboratory of Utilization and Conservation of Food and Medicinal Resources in Northern Region, Shaoguan Aromatic Plant Engineering Research Center, College of Biology and Agriculture, Shaoguan University, Shaoguan, China; ^2^ College of Horticulture, South China Agricultural University, Guangzhou, China

**Keywords:** clove basil, cadmium stress, transcriptomic profiles, Cd-responsive genes, phytoremediation

## Abstract

Planting aromatic plant might be a promising strategy for safely utilizing heavy metal (HM)-contaminated soils, as HMs in essential oil could be completely excluded using some special technologies with ease. Clove basil (*Ocimum gratissimum* L.) is an important aromatic plant used in essential oil production. Improving cadmium (Cd) tolerance in clove basil can increase its production and improve the utilization efficiency of Cd-contaminated soils. However, the lack of genomic information on clove basil greatly restricts molecular studies and applications in phytoremediation. In this study, we demonstrated that high levels of Cd treatments (0.8, 1.6 and 6.5 mg/L) significantly impacted the growth and physiological attributes of clove basil. Cd contents in clove basil tissues increased with treatment concentrations. To identify Cd stress-responsive genes, we conducted a comparative transcriptomic analysis using seedlings cultured in the Hoagland’s solution without Cd ion (control) or containing 1.6 mg/L CdCl_2_ (a moderate concentration of Cd stress for clove basil seedlings). A total of 104.38 Gb clean data with high-quality were generated in clove basil under Cd stress through Illumina sequencing. More than 1,800 differential expressed genes (DEGs) were identified after Cd treatment. The reliability and reproducibility of the transcriptomic data were validated through qRT-PCR analysis and Sanger sequencing. KEGG classification analysis identified the “MAPK signaling pathway,” “plant hormone signal transduction” and “plant-pathogen interaction” as the top three pathways. DEGs were divided into five clusters based on their expression patterns during Cd stress. The functional annotation of DEGs indicated that downregulated DEGs were mainly involved in the “photosynthesis system,” whereas upregulated DEGs were significantly assigned to the “MAPK signaling pathway” and “plant-pathogen interaction pathway.” Furthermore, we identified a total of 78 transcription factors (TFs), including members of bHLH, WRKY, AP2/ERF, and MYB family. The expression of six *bHLH* genes, one *WRKY* and one *ERF* genes were significantly induced by Cd stress, suggesting that these TFs might play essential roles in regulating Cd stress responses. Overall, our study provides key genetic resources and new insights into Cd adaption mechanisms in clove basil.

## Introduction

Heavy metals (HMs) are harmful environmental elements that are continuously released into the environment as a result of human and industrial activities (Jiang et al., 2022). The proliferation of HMs into the environment will cause soil and water contamination. Mining activity is one of most important reasons for HM pollution. For example, gold mining and processing are dominant industries in the Anka area of Nigeria. Such activities have caused a significant increase in HMs loading in soils and sediments in the local area (Adewumi and Laniyan, 2020). HMs-polluted soils lead to serious environmental issues and pose a threat to the safety of agricultural products (Zhu et al., 2018). Because most HMs could be absorbed by plants and stored in their sink tissues, eventually entering the food chain, and thereby posing a direct threat to human health (Caicedo-Rivas et al., 2022). To ensure the safety of agricultural products, the government typically imposes a moratorium on the use of HMs-polluted lands before the soil is completely remediated. This leads to a large amount of land being left wasted and idle, as it is reported that over 20% of farmlands are polluted by HMs in many countries, especially in developing countries (Xie et al., 2021). Therefore, the remediation or safe utilization of HMs-contaminated soils is important for limited farmland resources.

Remediation of HMs-contaminated soils mainly uses physical and chemical methods, such as immobilization, soil washing, chemical leaching, and stabilization (Faizan et al., 2022). These methods aim to remove HMs from the soil, or to reduce HMs concentration to a safe level, or to inactivate HM mobility. Nevertheless, these methods are generally expensive or detrimental to soil properties in practice (Zhu et al., 2018). Therefore, alternative and sustainable methods need to be developed. Phytoremediation is a biological process that uses plants to remove or enrich HMs from the soil (Simmer and Schnoor, 2022). However, the use of most hyper-accumulating plants, such as *Sedum alfredii* Hance, tall fescue and some plant species in the *Brassicaceae* and *Crassulaceae* families, has been limited due to their lack of economic value and small biomass as well as the long time required for remediation (Roosens et al., 2008; Gao et al., 2013; Dong et al., 2019). One potential strategy to address this issue is the remediation and simultaneous utilization of HMs-contaminated soils using some cash plants that can accumulate HMs in their tissues without suffering from toxicity.

Cultivating non-edible plants with high economic values for industrial purposes may be a feasible strategy to both safely utilize and remediate HMs-contaminated soils (Wang et al., 2023). Planting industrial plants can reduce the risk of human exposure to HMs through the food chain. Moreover, these plants can also have remediation effects on HMs-polluted soils by absorbing and accumulating HMs from the soil. Aromatic plants are cash crops used for essential oil extraction (Saeed et al., 2014), an important material in various product productions (Ekiert et al., 2021). Furthermore, HMs in essential oil can be readily removed through several special methods such as cold pressing and steam distillation (Gautam and Agrawal, 2017). Studies have reported that several aromatic plant species, such as peppermint, vetiver, and lemon grass, are highly tolerant to HMs, and HM stress could increase essential oil percentage and the synthesis of other metabolites, such as phenolic compounds (Pandey et al., 2019; do Prado et al., 2022; Wang et al., 2023). These reports suggest that planting aromatic plants is a sustainable and suitable method to utilize HMs-contaminated soils due to the monetary benefits.

Cadmium (Cd) is one of the most dangerous pollutants among HMs in nature and is widespread in soils (Zhang et al., 2023). As a toxic metallic element, Cd ions have been shown to negatively impact plant growth, even at very low concentrations (Jiang et al., 2020), resulting in reduced biomass and essential oil yield. Therefore, improving the Cd tolerance of aromatic plants through genetic engineering is crucial for the effective utilization of Cd-contaminated soils. Clove basil (*Ocimum gratissimum*) is an important species in the *Lamiaceae* family, because it produces essential oil in shoots and leaves (Ugbogu et al., 2021). Clove basil is a perennial shrub distributed in tropical and subtropical regions worldwide and can grow up to 1–3 m in height (Parasar et al., 2021). Due to its strong adaptability and resilience, it can grow in various soil types (Vilanova et al., 2018). However, to our best knowledge, available studies on how Cd stress influences clove basil performance are rare (do Prado et al., 2022). Furthermore, genomic data from *Ocimum* species are lacking, and available transcriptomic data is very limited (Parasar et al., 2021), with fewer than 150 ESTs deposited in the NCBI database. The lack of sufficient genomic data significantly hinders molecular studies in this plant species.

For plant species without sequenced genomes, RNA-seq is a relatively low-cost, simple, and efficient method to gather functional genomic data (Agarwal et al., 2014). This technique has been widely used to investigate transcriptomic responses to abiotic stress in several aromatic plants, such as peppermint (Wang et al., 2023) and *Artemisia annua* (Vashisth et al., 2018). We aim to investigate the effects of Cd treatment on the growth performance of clove basil seedlings and to present the first transcriptome profiles of clove basil leaves under Cd stress conditions at a global level using Illumina sequencing technology combined with bioinformatics analysis. Thus, the goals of this study are: 1) to investigate the influences of Cd stress treatments on growth and physiological attributes of clove basil; 2) to conduct transcriptomic profiles to a genome-wide extent; 3) to identify key genetic resources from clove basil under Cd stress for future molecular studies. Additionally, the functional analysis of Cd-responsive genes could provide valuable insights into the adaption mechanisms under Cd stress in plants.

## Materials and methods

### Materials, seedling cultivation and Cd stress treatment

The experimental setup was shown in Supplementary Figure S1. Clove basil (*Ocimum gratissimum*) seeds were sown in seedling-raising plates of matrix mixed with peat and perlite in a proportion 1:1. The seedlings, which had been grown for 14 days after germination and reached a height of approximately 8 cm, were individually transplanted into seedling-raising plates filled with a mixed matrix, ensuring ample space for further growth (each hole contained one seedling). After 21 days of growth in these plates, the seedlings were then transplanted into cuboid plastic containers filled with Hoagland’s nutrient solution. Prior to the Cd treatment, the seedlings were acclimatized in the nutrient solution for a period of 1 week (7 days). Each container contained 20 lines of seedlings and regarded as one biological replicate. All seedlings were grown in a greenhouse at Shaoguan university, Shaoguan, China, under a 16/8 h light/dark photoperiod and 23°C.

Different dosages of CdCl_2_ (Aladdin, China) solutions were separately added to the Hoagland’s nutrient solution (Supplementary Figure S1). The final concentrations of Cd ions in each treatment were as follows: 0 (control), 0.4, 0.8, 1.6, and 6.5 mg/L for Cd treatments, respectively. In a previous study, we investigated the effects of different intensities of Cd treatments on peppermint growth (Wang et al., 2023), as both peppermint and clove basil belong to the *Labiatae* family. Therefore, in this study, we designed the Cd concentration of stress treatments based on our previous study to test Cd responses in clove basil seedlings. Each treatment group contained 60 seedling lines (three biological replicates).

During Cd treatment, leaf, shoot and root samples of clove basil seedlings were collected separately at 0 h, 24 h (1 day), 72 h (3 days) and 14 d (2 weeks) for further measurements or investigations. For sample collection, the samples from nine lines of one treatment were combined into a sample. After sampling, samples were quickly frozen within liquid nitrogen and stored at −80°C.

### Determination of Cd contents in clove basil seedlings

The collected clove basil samples (leaf, shoot, and root) were dried in an air-circulating oven at 105°C for 10 min and at 80°C until a constant weight was reached. Then, the samples were ground into powder and sieved through a 100 mesh sieve. 200 mg of powders were digested with 10 mL of high-purity nitric acid-perchloric acid solutions (4:1, v/v). Finally, the digested solutions were added up to 100 mL using 2% nitric acid. Cd contents in clove basil samples were measured using a flame atomic absorption spectrometer (A3-AFG, PERSEE, China). The samples were grouped into roots, shoots, and leaves for determining Cd concentrations, respectively. The result of Cd content was expressed as milligram per kilogram dry weight (mg kg^−1^ DW) (Gao et al., 2013).

### Evaluation of the effects of Cd treatments on the growth of clove basil seedlings

Phenotypic changes of clove basil under different intensities of Cd treatments were analyzed first to test their Cd tolerance before RNA-Seq. The effects of different concentrations of Cd treatments on the seedling growth of clove basil were evaluated at 14 days after Cd treatment. The height of the seedlings was measured using a scale ruler, and the seedlings were dried to a constant weight and then used for weight measurements.

Chlorophyll relative contents (SPAD) in leaves under different levels of Cd stress were measured using a portable plant nutrient analyzer (TYS-4N, China). The contents of chlorophyll pigments in leaves of clove basil were determined using the method developed by Lichtenthaler (1987) through a UV spectrophotometer (UV-6100, SHjingmi, China). The root system of clove basil seedling was pictured and analyzed with a MICROTEK scanner (MRS-9600 TFU2L, China) (Wang et al., 2023).

### Analysis of physiological parameters of clove basil under Cd stress

The activities of superoxide dismutase (SOD), catalase (CAT), and peroxidase (POD) in clove basil leaves were measured using a previously described method (Wang et al., 2023; Yuan et al., 2023). Total soluble protein (TSP) content in clove basil leaves was determined with the Coomassie brilliant blue method (Snyder and Desborough, 1978).

### Total RNA extraction

The total RNA from clove basil leaves was isolated using an RNAprep Pure Plant Plus Kit (Tiangen, China) (Xiao et al., 2021). RNA quantification and qualification were performed as previously described (Wang et al., 2022).

Unlike other Cd-accumulating plants that lack economic value, clove basil is commonly used for extracting essential oil, which is primarily produced and stored in the leaves. Furthermore, the transport of Cd ions to the cytoplasm and vacuole of plant leaves through HM transporters is one of significant mechanisms for Cd detoxification. One of our objectives is to explore potential mechanisms of Cd detoxification in clove basil, a commercially valuable plant that produces essential oil, which might differ from those in other plants under Cd stress. Therefore, we focused our investigations on the transcriptome responses of clove basil leaves in response to Cd stress in this study.

### cDNA library construction and RNA sequencing

The preparation of cDNA library was carried out using a NEB Next Ultra RNA Library Prep Kit for Illumina (NEB, United States) following the manufacturer’s recommendations at the Biomarker Biotechnology Corporation (Beijing, China). The detailed instructions for library construction were described in a previous study (Wang et al., 2021a). The quality of all cDNA libraries was assessed using a Bioanalyzer 2100 system (Agilent Technologies, United States). Before transcriptome sequencing, a cBot Cluster Generation System with TruSeq PE Cluster Kit v4-cBot-HS (Illumia, United States) was employed to generate clusters. Finally, all constructed libraries were sequenced using the Illumina HiSeq2500 system, and paired-end reads were generated and converted to fastq format using bcl2fastq tool (Illumina, United States).

### Transcriptomic data analysis and the identification of differential expressed genes (DEGs)

The raw reads were processed first through in-house perl scripts to obtain clean reads by removing those containing adapters and ploy-N. At this step, GC-contents, Q20, Q30, and sequence duplication level in the clean data were calculated. We used Trinity software for transcriptome assembly (Zhu et al., 2018).

Expectation maximization tool was used to quantify the expression values of assembled transcripts, and we calculated fragments per kilobase of transcript per million fragments mapped (FPKM). To compare the differences in expression levels between two tested groups (each consisting of three replicates), we used the DESeq2 package. Differential expressed genes (DEGs) were identified based on the following thresholds: Q-value (adjusted *p*-value) ≤ 0.01 and fold change (FC) ≥ 1.5 (Wang et al., 2023). Each treatment had three biological replicates. Transcriptomic data was compared at specific time points to screen for DEGs: treatment at 24 h versus control at 24 h, and treatment at 72 h versus control at 72 h. FPKM values were utilized to demonstrate the expression patterns of DEGs during Cd treatment at different time intervals (0, 24, and 72 h).

### Functional annotation and classification of the detected DEGs

The assembled sequences were searched against the NR protein database in the NCBI (ftp://ftp.ncbi.nlm.nih.gov/blast/db/) using BLASTX algorithm. Other public databases including Gene Ontology (GO) (http://www.geneontology.org/), Kyoto Encyclopedia of Genes and Genomes (KEGG) (http://www.genome.jp/kegg), Swiss-Prot (http://www.uniprot.org/), Pfam (https://www.ebi.ac.uk/interpro), and KOG/COG (http://www.ncbi.nlm.nih.gov/COG/) were also employed to annotate gene function or classification.

### The quality validation of transcriptomic data by qRT-PCR analysis and Sanger sequencing

To validate the expression patterns of DEGs during Cd stress, we employed qRT-PCR analysis, and nine representative DEGs involved in Cd stress response were randomly selected. qRT-PCR analysis was conducted using previous methods (Xiao et al., 2022). The relative expression level of a specific gene was calculated with the 2^−ΔΔCT^ method developed by Schmittgen and Livak (2008). An annotated gene known as *Actin 7* (c52716.graph_c0) was used as an internal control gene to perform normalization in this study. The expression levels of this *Actin* gene were not significantly influenced by Cd treatment (Supplementary Figure S2), indicating that it could be used as an internal control gene. The results of the expression validation of DEGs also demonstrated the reliability and reproducibility of this *Actin* gene as an internal reference gene (Figure 4).

Five representative DEGs were selected for PCR amplification and Sanger sequencing. First, the full-length sequence of each gene was cloned into pUCm-T vectors. Subsequently, the recombinant vectors were transformed into *E. coli* (DH-5α). The positive clones were validated again by PCR amplification, and then sequenced through Sanger sequencing. The sequences were analyzed using the BLAST program for identity alignment of assembled transcripts with Sanger sequences.

Specific primers used in this study were designed with an online tool, Primer-BLAST (https://www.ncbi.nlm.nih.gov/tools/primer-blast/index.cgi), and listed in Supplementary Table S1.

### Statistical analysis

The data in this study were expressed as the mean ± standard deviation (SD), and values were calculated with the Excel 2016 software (Microsoft, United States). To analyze statistical differences between different samples, we used SPSS 22.0 software (IBM, United States) and employed a Student’s t-test with *p* ≤ 0.05 considered statistically significant. The statistical differences among samples were indicated with lowercase letters above bars.

## Results

### Effects of Cd treatment on the growth of clove basil

**FIGURE 1:**
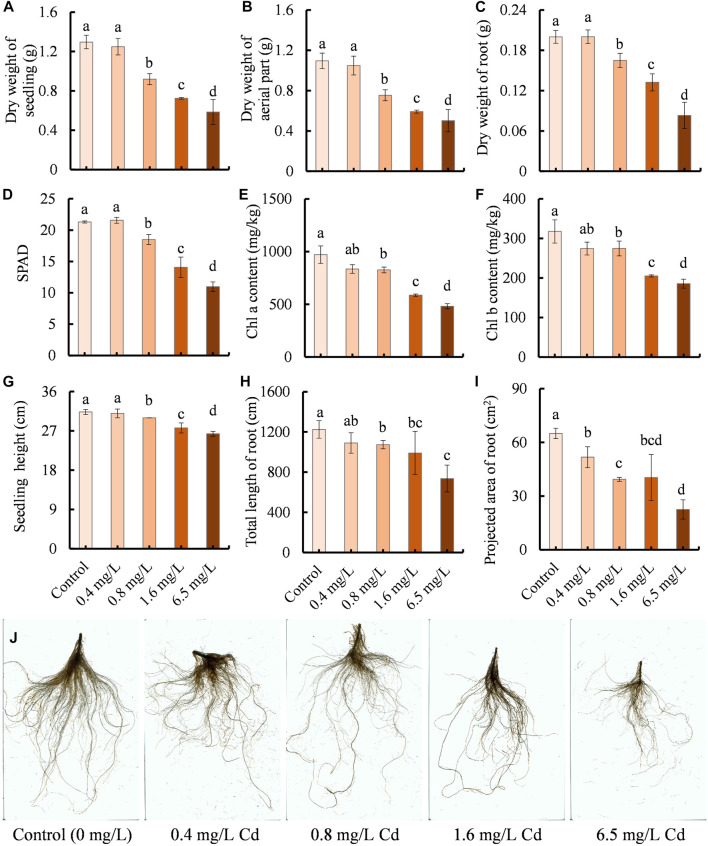
Effects of Cd treatments with different concentrations (0, 0.4, 0.8, 1.6, and 6.5 mg/L) on the growth performance of clove basil. **(A)**, dry weight of seedlings (whole plant); **(B)**, dry weight of aerial part; **(C)**, dry weight of root; **(D)**, relative contents of chlorophyll (SPAD); **(E,F)**, contents of chlorophyll a and b pigments, respectively; **(G)**, seedling height; **(H)**, total length of root; **(I)**, projected area of root; **(J)**, root morphology. The growth performance of clove basil seedlings was evaluated following 2 weeks (14 days) of Cd treatments. Each value is presented as means ± standard error from three repeats (*n* = 3). Statistical differences (*p* ≤ 0.05) between treatments were compared using the SPSS software and indicated using different letters above the bars.

The growth of clove basil was evaluated under different concentrations of Cd treatments by measuring dry weight, chlorophyll contents, seedling height, and root development. Cd treatments led to significant reductions in dry weight, chlorophyll contents, height, and root system development of clove basil (Figure 1). Compared with the control group, seedlings exposed to Cd concentrations exceeding 0.4 mg/L had decreased values ranging from 4.26% to 65.31% (Figure 1). High levels of Cd treatments caused severe Cd injures on young leaves (Supplementary Figure S3).

No significant differences were observed between the control and 0.4 mg/L Cd treatment in terms of dry weight, chlorophyll contents, seedling height, and root length (Figures 1A–I). Furthermore, no visible differences in the root morphology were found between the control and 0.4 mg/L Cd treatment and no Cd injuries on young leaves were found in the control and 0.4 mg/L Cd treatment (Figure 1J; Supplementary Figure S3). These results suggest that clove basil has the potential to withstand mild Cd stress, making it useful for Cd-contaminated soil utilization.

### The contents of Cd ions in clove basil tissues

**FIGURE 2:**
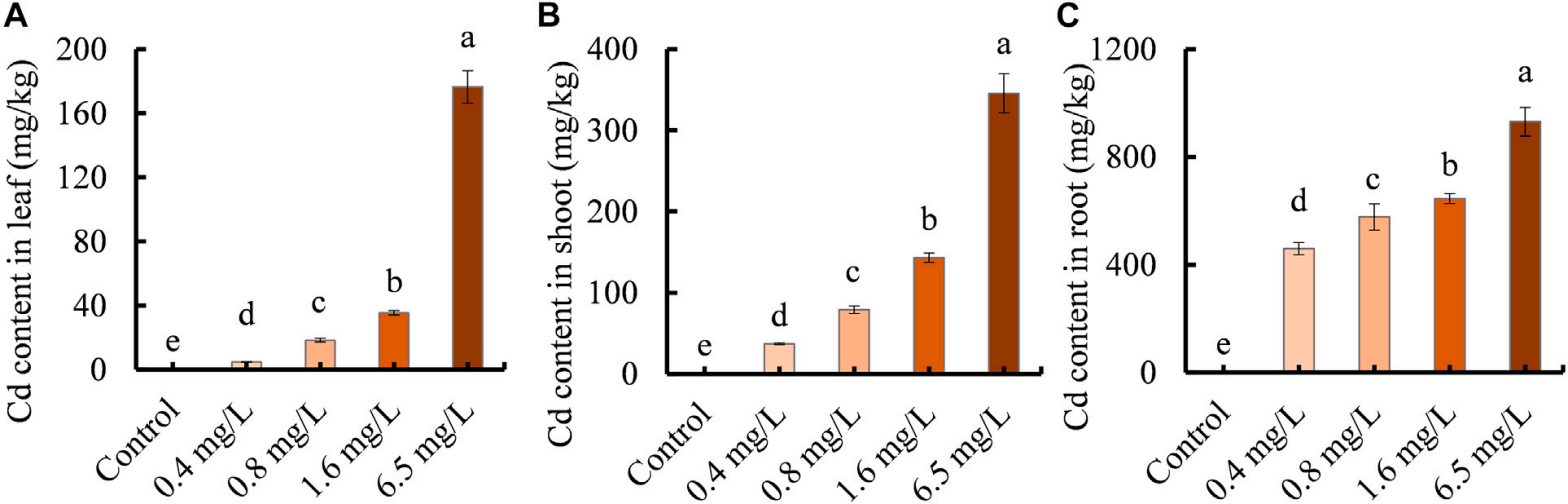
Cd ion contents in clove basil tissues following 2 weeks of Cd treatments (0, 0.4, 0.8, 1.6, and 6.5 mg/L). **(A)**, Cd ion contents in leaves; **(B)**, Cd ion contents in shoots; **(C)**, Cd ion contents in roots. Each value is presented as means ± standard error from three repeats (*n* = 3). Statistical differences (*p* ≤ 0.05) between treatments were compared using the SPSS software and indicated using different letters above the bars.

We investigated the ability of clove basil seedlings to accumulate or enrich Cd ions by measuring Cd ion contents in leaves, shoots, and roots following treatment with different concentrations of CdCl_2_. No Cd ions were detected in the control group tissues. Cd ion contents in all tissues increased with increasing Cd treatment concentration, with the highest Cd accumulation observed in leaf, shoot, and root tissues at 6.5 mg/L Cd treatment (Figure 2). Notably, root tissues had significantly higher Cd contents than leaf and shoot tissues under various Cd treatments (Figure 2), suggesting that clove basil accumulated much less Cd ions in the above ground part.

### RNA-seq data analysis and the identification of DEGs under Cd stress

In order to investigate physiological and molecular changes of clove basil under Cd stress, a moderate stress concentration (1.6 mg/L) was selected to treat clove basil seedlings for RNA-seq analysis. A total of 15 cDNA libraries including 5 treatment groups (control at 0, 24 and 72 h, Cd treatment at 24 and 72 h, three biological replicates) were constructed. The average number of pair-end raw reads among the 15 samples was 22,147,978 (Supplementary Table S2). Quality evaluation of 15 samples generated 104.38 Gb data and the mean size of 15 cDNA libraries was 6.96 Gb. After filtration, GC contents of all samples varied from 48.72% to 50.81%, and Q30 values varied from 92.64% to 93.88% (Supplementary Table S2). The length of 76.88% transcripts was over 500 bp, and more than 58.00% transcripts were over 1 kb (Supplementary Table S3). Transcriptome assembly generated 79,637 transcripts (Supplementary Data Sheet S1) and 51.56% (41,060) unigenes were successfully annotated against nine public databases (Supplementary Table S4). Mapping efficiency of 15 samples varied from 75.34% to 77.27% (Supplementary Table S5). The results indicated that these high-quality transcriptome data can be further used for bioinformatics analysis.

**FIGURE 3:**
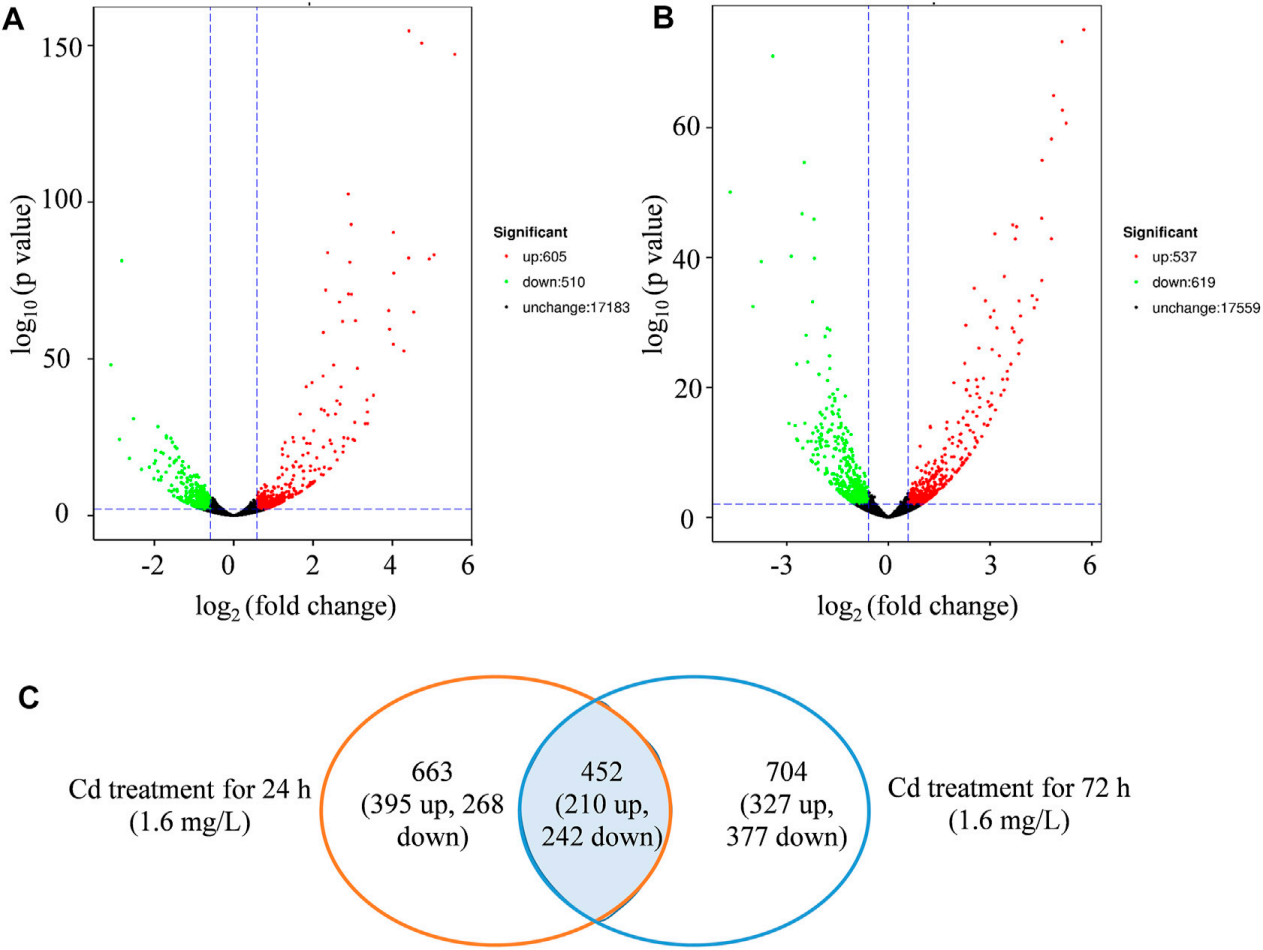
The identification of differential expressed genes (DEGs). **(A)**, volcano map of DEGs regulated by Cd treatment (1.6 mg/L) at 24 h. **(B)**, volcano map of DEGs regulated by Cd treatment (1.6 mg/L) at 72 h. **(C)**, venn diagram of DEGs regulated by treatment (1.6 mg/L) in the comparison between 24 and 72 h.

The expression differences of specific gene were compared to the control at each sampling point (24 and 72 h, respectively) (Figure 3; Supplementary Data Sheets S2, S3). The DEGs were divided into up or downregulated transcripts based on regulation manner. Totally, 1,115 (605 up and 510 downregulated) DEGs were identified in Cd-treated leaves compared with control groups at 24 h time point (Figure 3A), while 1,156 (537 up- and 619 downregulated) were found at 72 h (Figure 3B). Cd treatment induced more DEGs at 24 h of initial stage, whereas more DEGs were repressed at 72 h (Figure 3C).

At 24 h, Cd treatment specifically affected the expressions of 663 DEGs (395 up and 268 downregulated), while at 72 h, 704 DEGs (327 upregulated and 377 downregulated) were specifically affected (Figure 3C). Additionally, 452 overlapped genes (210 up and 242 downregulated) were differentially expressed at both time points (Figure 3C). The overall expression profile of these 452 DEGs was shown in Supplementary Figure S4, with most showing remarkable differences between Cd treatment and control.

### The quality evaluation of transcriptomic data

**FIGURE 4:**
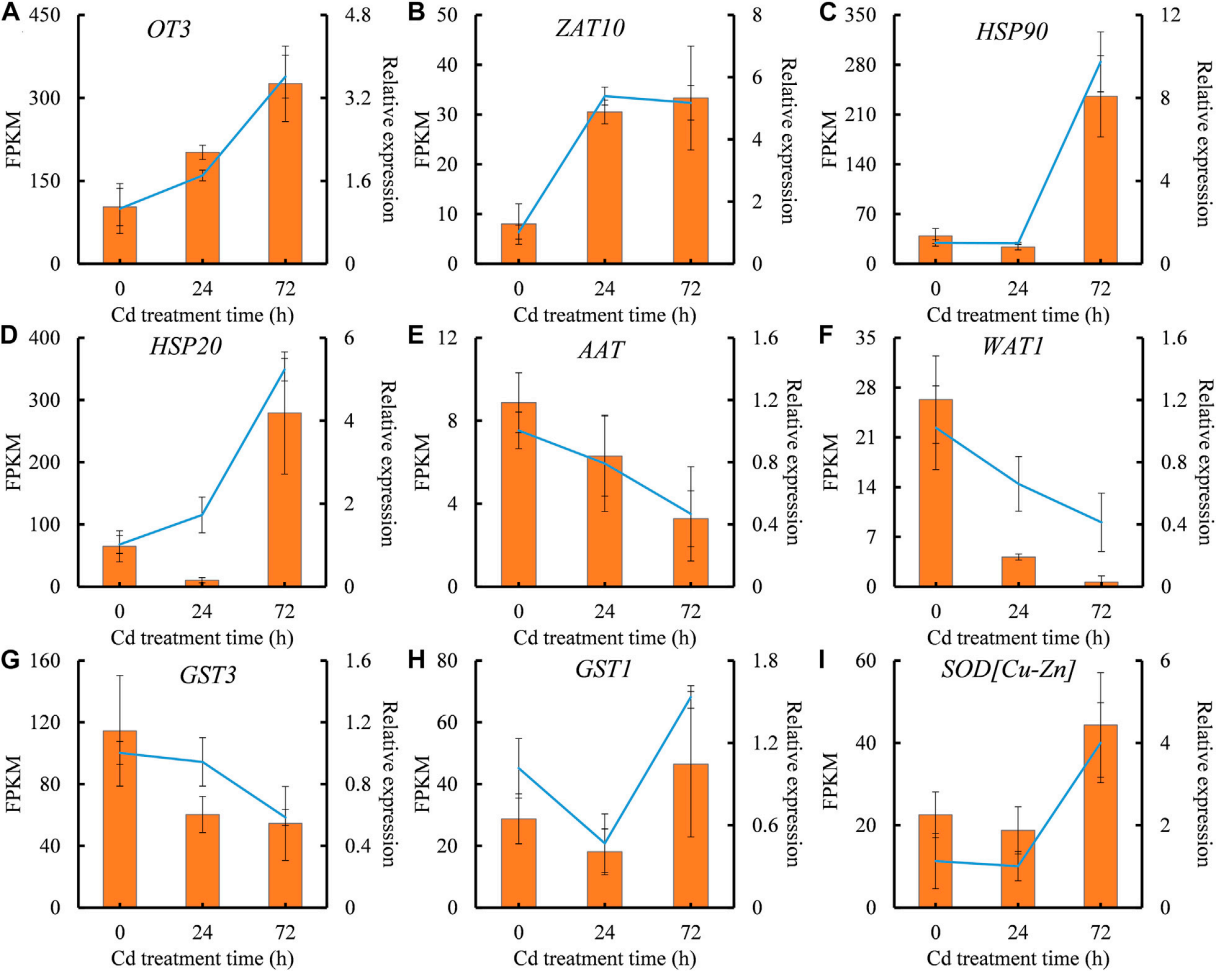
The expression patterns of representative differential expressed genes (DEGs) validated by qRT-PCR analysis. **(A)**, *OT3* expression. **(B)**, *ZAT10* expression. **(C)**, *HSP90* expression. **(D)**, *HSP20* expression. **(E)**, *AAT* expression. **(F)**, *WAT1* expression. **(G)**, *GST3* expression. **(H)**, *GST1* expression. **(I)**, *SOD[Cu-Zn]* expression. The X-axis represents treatment time. The Y-axis on the left represents the expression level of specific gene obtained from RNA-seq (histogram), and on the right, from qRT-PCR analysis (line chart). Each value is presented as means ± standard error from three repeats (*n* = 3). Standard errors are indicated in figures using the bars.

qRT-PCR analysis, PCR amplification and Sanger sequencing of random genes were carried out to verify the reproducibility and credibility of RNA-seq data. Four DEGs (*OT3*, *ZAT10*, *HSP90*, and *HSP20*) showed steady increase, while three DEGs (*AAT*, *WAT1*, and *GST3*) exhibited constant reduction during Cd treatment in RNA-seq data. The expressions of two DEGs, *GST3*, and *SOD[Cu-Zn]*, were only induced by Cd treatment at 72 h (Figure 4). qRT-PCR validation of these nine representative DEGs showed high similarity with RNA-Seq data (Figure 4), indicating that the transcriptomic data could really, instantly reflect transcriptomic changes under Cd stress.

The ORF sequences of five DEGs were successfully amplified through PCR and sub-cloned to T vectors for Sanger sequencing, which matched closely to RNA-seq data with only a few bases not exactly matching (Supplementary Figure S5). This provides further evidence for the accuracy and reliability of the transcriptome sequencing data.

### Functional annotation of Cd-responsive genes with COG and GO database

Phylogenetic classification was performed using Orthologous Groups (COG) analysis. A total of 392 DEGs generated by Cd treatment for 24 h were matched and grouped into 20 functional classes, with “signal transduction mechanisms” (43, 10.97%) and “carbohydrate transport and metabolism” (37, 9.44%) being the largest groups in percentage (Supplementary Figure S6A; Supplementary Data Sheet S2). Similarly, 460 DEGs from Cd treatment for 72 h were matched and classified into 22 functional classes, with “general function prediction only” (49, 10.65%), “carbohydrate transport and metabolism” (44, 9.57%), and “posttranslational modification, protein turnover, chaperones” (42, 9.13%) being the three largest groups in percentage (Supplementary Figure S6B; Supplementary Data Sheet S3).

The GO database were also used to classify the potential functions of annotated DEGs. Cd-treatment-caused DEGs were assigned to 45 functional groups under three major categories, showing similar classifications at both 24 h and 72 h (Supplementary Figure S7). In the biological process category, “metabolic process” and “cellular process” had most DEGs assigned. In the cellular component category, the terms of “cell,” “cell part,” “membrane,” “membrane part and organelle” were dominant terms, while “binding” and “catalytic activity” were the most common clusters in the molecular function category (Supplementary Figure S7).

### The KEGG pathway analysis and expression patterns of Cd-responsive genes in clove basil

**FIGURE 5:**
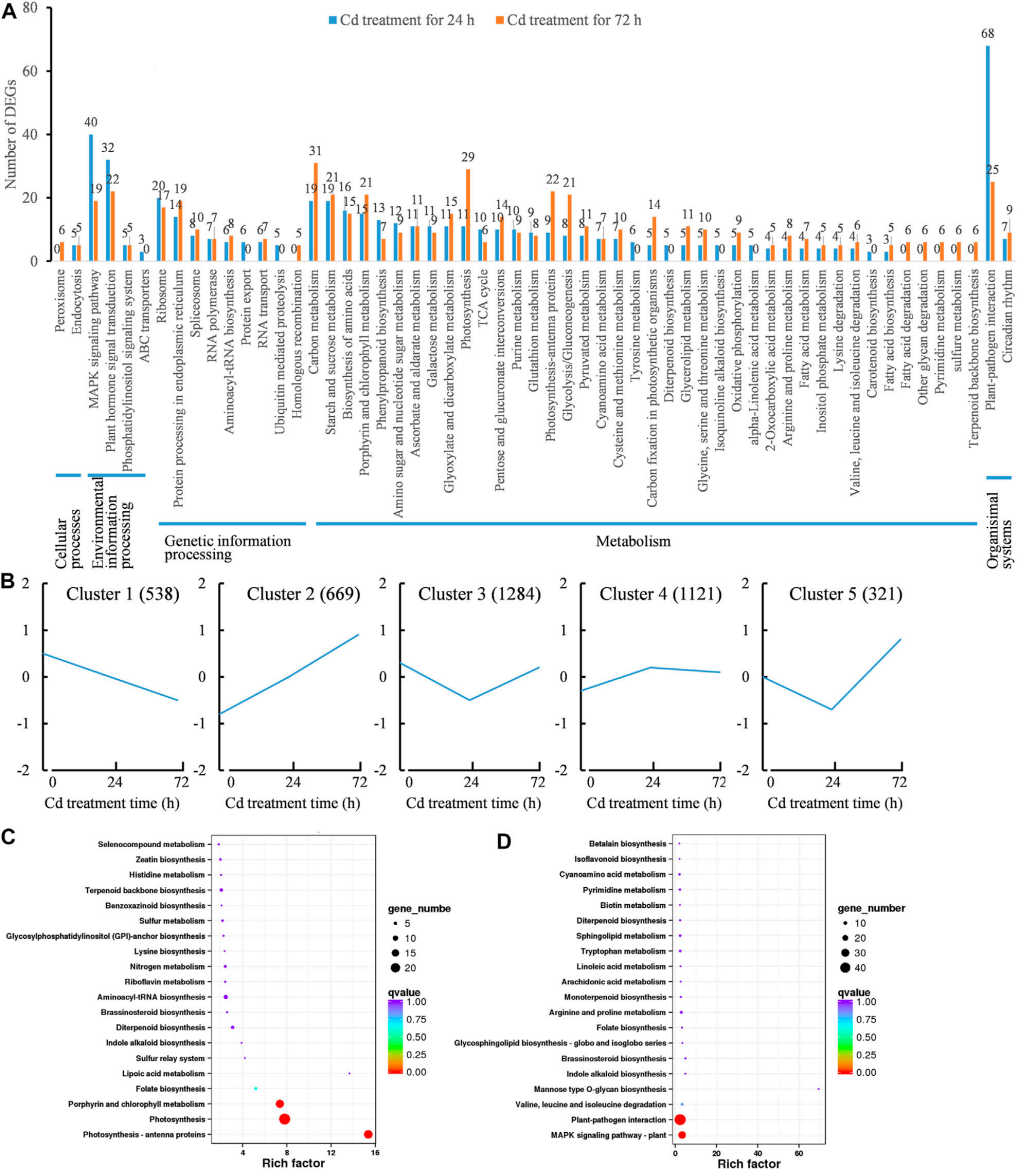
Functional annotation and expression patterns of differential expressed genes (DEGs) caused by Cd treatment (1.6 mg/L). **(A)**, KEGG classification analysis of DEGs. **(B)**, expression patterns of DEGs during 72 h of Cd treatment. **(C)**, KEGG enrichment analysis of DEGs from the Cluster 1; **(D)**, KEGG enrichment analysis of DEGs from the Cluster 2.

KEGG pathway analysis was further performed to predict the functions of Cd-responsive genes. A total of 519 DEGs were assigned to 50 specific KEGG pathways at 24 h, while 555 DEGs were assigned to 53 KEGG pathways at 72 h (Figure 5A). At 24 h, the “plant-pathogen interaction pathway” had the most DEGs assigned (68, 13.10%), followed by the “MAPK signaling pathway” (40, 7.71%) and the “plant hormone signal transduction” pathway (32, 6.17%). At 72 h, most DEGs were classified into pathways of “carbon metabolism” (31, 5.59%) and “photosynthesis” (29, 5.23%) (Figure 5A). The results implied that long term of Cd exposure damaged photosynthetic system, which led to insufficient energy supply for survival and ultimately resulted in cell death under Cd stress.

The Cd-responsive genes were classified into five groups based on their expression patterns during Cd treatment, with 538 DEGs in cluster 1, 669 DEGs in cluster 2, 1,284 DEGs in cluster 3, 1,121 DEGs in cluster 4, and 321 DEGs in cluster 5 (Figure 5B). As Cd-induced impacts on clove basil were positive or negative, we focused on analyzing the functions of DEGs that were constantly induced (cluster 2 DEGs) or repressed (cluster 1 DEGs) through KEGG enrichment analysis.

DEGs in the cluster 1 showed significant downregulation in expression due to Cd stress and were enriched in three KEGG pathways: “porphyrin and chlorophyll metabolism” (17 genes), “photosynthesis” (24 genes) and “photosynthesis-antenna protein” (18 genes) (Figure 5C). This conforms that Cd stress significantly reduced photosynthetic efficiency in clove basil. On the other hand, upregulated DEGs (cluster 2) were significantly enriched in “plant-pathogen interaction” pathway (13 genes) and “MAPK signaling pathway” (8 genes) (Figure 5D). Previous studies observed similar mRNA accumulation related to pathogenesis/disease-related genes and MAPK signaling in Cd-treated roots of tall fescue (Zhu et al., 2018). These results indicate important crosstalk between pathogen stress signaling and HM signaling.

### Transcription factors involved in Cd stress response in clove basil

**FIGURE 6:**
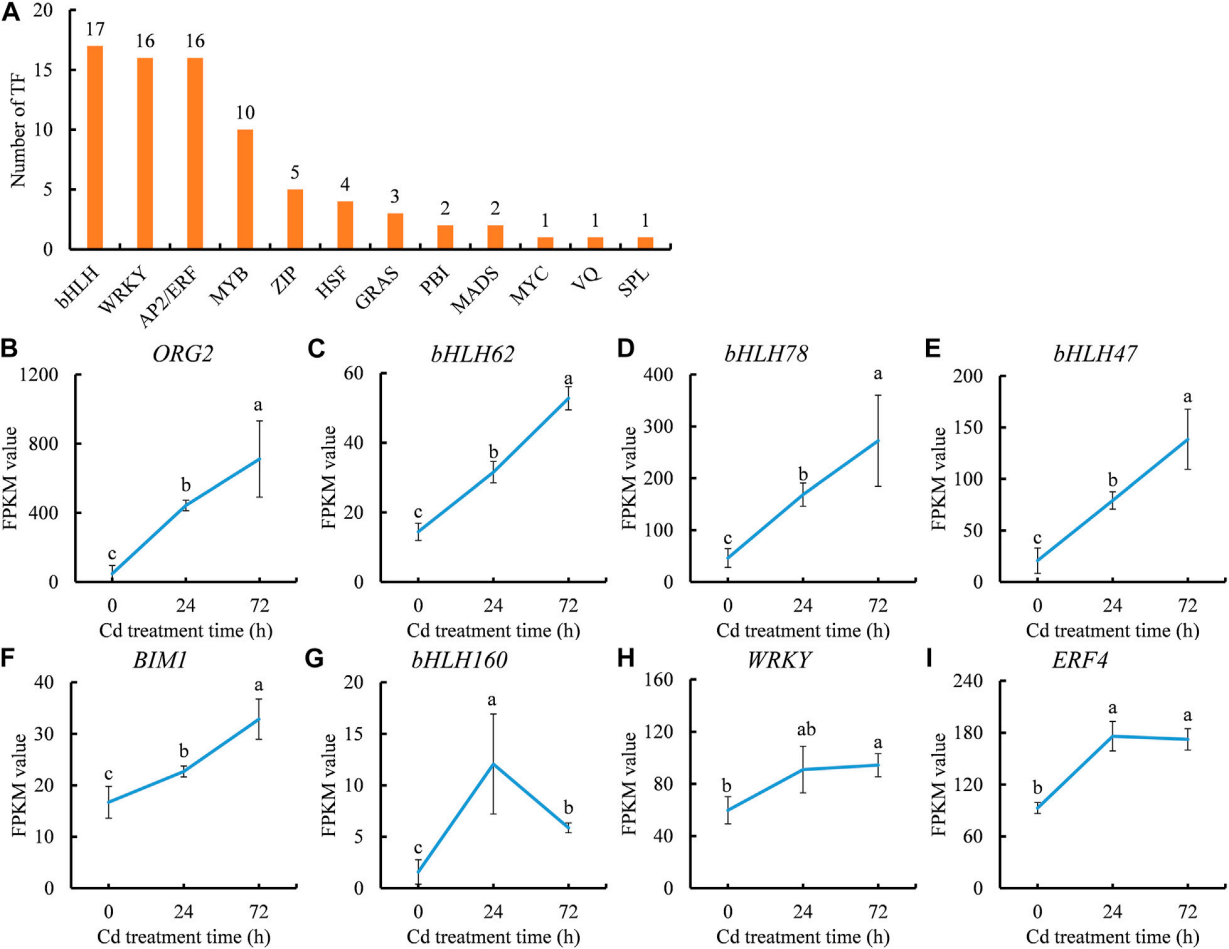
Cd stress responsive transcription factor (TF) genes **(A)** and expression trends of Cd stress inducible TF genes **(B–I)** during the period of Cd treatment. **(B)**, *ORG2* gene. **(C)**, *bHLH62* gene. **(D)**, *bHLH78* gene. **(E)**, *bHLH47* gene. **(F)**, *BIM1* gene. **(G)**, *bHLH160* gene. **(H)**, *WRKY* gene. **(I)**, *ERF4* gene. Each value is presented as means ± standard error from three repeats (*n* = 3). Statistical differences (*p* ≤ 0.05) between treatments were compared using the SPSS software and indicated using different letters above the bars.

Given that transcription factors (TFs) play important roles in regulating the expression of Cd-responsive genes, and therefore, it is necessary to identify Cd-inducible TFs. A total of 78 TFs that could respond to Cd stress were screened out, mainly belonging to 12 families including *bHLH*, *WRKY*, *AP2/ERF*, and *MYB* (Figure 6A). *bHLH* family members constituted a large proportion (17 out of 78) of Cd-responsive TFs, while a subset of Zinc finger family members, including the WRKY subfamily, were involved in responses to Cd stress. Relatively fewer TF members were characterized as MYB, HSF, GRAS, PBI, MADS, MYC, VQ, and SPL family members (Figure 6A).

Among the Cd-inducible TF genes, eight showed significantly increased expression after Cd treatment, with seven continually induced by Cd stress. Only one gene, *bHLH160* (c51250.graph_c0), was sharply induced at 24 h in the leaves of clove basil. Five of the seven Cd-inducible TF genes belonged to the *bHLH* family (c58694.graph_c0, c57229.graph_c0, c56571.graph_c1, c51522.graph_c0, and c62303.graph_c0), while one belonged to the *WRKY* family (c63519.graph_c0) and another belonged to the *AP2/ERF* family (c48938.graph_c0) (Figures 6B–I). These results suggest that *bHLH* TFs may be involved in the adaptation regulation of Cd stress, highlighting the importance of these five Cd-inducible *bHLH* genes as potential candidates for improving Cd tolerance in clove basil through genetic engineering.

### Effects of Cd treatment on the antioxidant system in clove basil

Cd toxicity in plants is mainly due to oxidative damages (Ahmad et al., 2021), hence, monitoring physiological indices related to oxidative stress in clove basil after Cd treatment is important. The contents of TSP, activities of SOD and POD in leaves were decreased after Cd treatment, although SOD activity did not show significant differences before and after Cd treatment (Supplementary Figures S8A–C). After 72 h of Cd treatment, POD activities and TSP contents were reduced by 8.52% and 64.72%, respectively, indicating that Cd stress might have repressed the biosynthesis and accumulation of new proteins or promoted protein degradation. CAT activity showed an overall increasing trend and was enhanced by 119.63% after Cd treatment (Supplementary Figure S8D).

Furthermore, the expression profiles of DEGs encoding antioxidant enzymes such as POD, SOD and CAT were analyzed during Cd treatment (Supplementary Figure S8E). Specific genes in the same family showed a significant expression pattern, suggesting that these antioxidant-related genes intricately regulated enzyme activity at the translation level. The changes in activity of these three antioxidant enzymes were validated at the transcriptional level using several representative genes during Cd stress (Supplementary Figures S8F–K). Cd treatment significantly downregulated the expression of two *POD* genes (Supplementary Figures 8F, G). The expression levels of two *SOD* genes did not show significant differences at 0 and 72 h (Supplementary Figures 8H, I). However, Cd treatment significantly induced the expression of two *CAT* genes (Supplementary Figures 8J, K), consistent with the increased enzyme activity. These results suggest that H_2_O_2_ scavenging activity was enhanced after Cd treatment, indicated by the significant increase in CAT activity, which may be a potential mechanism of clove basil adaptation to Cd stress.

The glutathione (GSH) biosynthesis pathway is positively related to phytochelatin (PC) synthesis, which helps in mitigating Cd-induced oxidative stress. Therefore, we analyzed the expression profiles of genes in the GSH pathway. During Cd stress, a total of 10 genes encoding glutathione S-transferase (GST) were differentially expressed. Among them, the expressions of four *GSTs* (c54510.graph_c0, c51495.graph_c4, c59296.graph_c0 and c51498.graph_c0) were initially reduced at 24 h, but then were strengthened at 72 h, whereas the expression levels (c52100.graph_c0, c45992.graph_c0, and c33411.graph_c0) were constantly increased during Cd treatment. The remaining three GSTs (c50364.graph_c0, c62802.graph_c0, and c55400.graph_c0) showed an overall decreased trend in expression after Cd treatment (Supplementary Figures S9).

Two DEGs encoding phytochelatin synthase (PCS) were identified, and their expression levels were significantly increased after long-term Cd treatment (72 h) compared to 0 h. The expression of a glutathione reductase (GR) gene was downregulated by Cd treatment. Cd treatment significantly regulated the expression of four glutathione peroxidase (GPX) genes in clove basil, with all expressions being repressed. Additionally, two genes involved in the biosynthesis pathway of GSH were significantly induced by Cd stress (Supplementary Figures S9). These results suggest that Cd stress influences GSH biosynthesis, and the increased GSH levels would help alleviate Cd-induced oxidative stress in clove basil.

## Discussion

### Clove basil has abilities to enrich Cd ions and can tolerate mild Cd stress

Cd is a non-essential element for plant growth and development, and is highly toxic to plants (Shaari et al., 2022). Cd exposure is believed to reduce plant biomass (Halim et al., 2020). As clove basil is a cash plant used for producing essential oil, its biomass positively correlates with the yield of essential oils. Therefore, it is important to determine how much Cd stress intensity influences the growth of clove basil seedlings for practical applications. In this study, we evaluated the effects of different dosages of CdCl_2_ treatments on the growth of clove basil and Cd ion contents in clove basil tissues. The results showed that Cd ion contents in stress treatments varied from 4.68 mg/kg in leaves of 0.4 mg/L Cd group to 931.11 mg/kg in roots of 6.5 mg/L Cd group (Figure 2), implying that clove basil seedlings had the ability to accumulate or enrich Cd ions in inner tissues. However, low-level Cd treatment (0.4 mg/L) did not reduce the growth and biomass of clove basil plant (Figure 1). These results demonstrated that clove basil might be an alternative plant for the utilization and remediation of mildly Cd-contaminated soils. The application of clove basil in remediating Cd-contaminated soils can reduce the safety risk of agricultural products while enhancing the utilization efficiency of HM-contaminated soils. To our best knowledge, this study is the first report on investigations of Cd enrichment ability in clove basil.

Cd accumulation in plants shows obvious tissue-specificity, with Cd toxicity showing a dose-effect (Jin et al., 2018). The highest accumulation of Cd ions is in roots followed by shoots and leaves in *Polygonatum sibiricum*, a traditional Chinese medicinal herb (Xie et al., 2021). Our study confirms that in all Cd treatments, leaves had the lowest Cd contents while roots contained the highest Cd contents (Figure 2), which benefits the safe utilization of Cd-contaminated soils, as essential oil is mainly generated and stored in leaves. High levels of Cd stress inhibit plant growth and damage the photosynthesis system of mung bean (Aqeel et al., 2021). Similarly, we observed that the growth performance of clove basil seedlings was significantly reduced by high levels of Cd treatments (0.8, 1.6, 6.5 mg/L), and the chlorophyll pigment contents in clove basil leaves were also reduced following Cd treatments (Figure 1). These results suggest that high degrees of Cd stress negatively affect the growth and biomass of clove basil, which could have implications for the essential oil yield of this important aromatic plant. Therefore, identification of key Cd-responsive genes that have important roles in the tolerance of Cd stress or in the regulation of Cd response is necessary for molecular breeding of clove basil tolerant to moderate and even severe Cd stress.

### Transcriptomic data evaluation and functional analysis of Cd-responsive genes

The above results demonstrate the potential of clove basil to safely utilize and remediate Cd-contaminated soils. However, the lack of available genomic data for clove basil greatly restricts molecular studies of this species (Parasar et al., 2021). The development of nucleic acid sequencing technologies has made it possible to use RNA-seq combined with bioinformatics analysis to reveal plant responses to Cd stress at the molecular level and screen key genes that could positively regulate Cd tolerance in plants. In this study, we sequenced the whole transcriptome of clove basil under Cd stress conditions using Illumina HiSeq technology.

The quality of RNA-seq data is crucial for reliable analysis results. In this study, over 51.00% of transcripts were successfully annotated (Supplementary Table S4), which was higher than other plant species without reference genomic data, such as 40.38% for *Crossostephium chinensis* (Yang et al., 2017), 46.60% for peppermint plant (Wang et al., 2023), and 35.92% for wild paper mulberry (Xu et al., 2019). The expression patterns of representative DEGs from RNA-seq were highly consistent with those detected by RNA-seq (Figure 4). These results suggest that both the quality of transcriptomic data and the mapping effectiveness were high. Sanger sequencing also confirmed the high accuracy of transcriptomic data (Supplementary Figure S5).

Cd stress alters the expression levels of certain genes in plants, known as Cd-responsive genes (Zhao et al., 2021). However, the expression pattern of Cd-responsive genes and the regulation intensity vary between plant species. For example, in *Populus × canadensis* “Neva” leaves, it was reported that 2,816 genes were Cd-responsive, and 1,346 (47.80%) decreased in abundance (Li et al., 2021). In the roots of tall fescue, a total of 2,594 Cd-responsive genes were detected, while only 52 DEGs were found in the leaves (Zhu et al., 2018). Our study found that Cd treatment significantly regulated the expression of over 1,800 DEGs in clove basil leaves, with the numbers of downregulated and upregulated DEGs being almost equal (Figure 3). These results demonstrate that plant responses to Cd stress vary greatly among different species and organs. Investigating the Cd stress response in clove basil would provide new insights for understanding the Cd adaptation mechanisms of plants.

The functions of the DEGs were subsequently annotated through GO, COG, and KEGG classification. In this study, the downregulated DEGs were found to be significantly enriched in the photosynthesis pathway (Figure 5C). Additionally, seedling treated with Cd stress led to a significant reduction in SPAD values and chlorophyll contents in clove leaves (Figure 1). Furthermore, Cd treatment resulted in high Cd accumulations in leaves (Figure 2) and caused severe injuries to young leaves of seedling under Cd stress (Supplementary Figure S3). These results together suggest that Cd stress induces damages to the photosynthesis system, which could explain the observed retardation in the growth of clove basil seedlings. Therefore, implementing some agronomic technologies to enhance photosynthetic efficiency could be a viable approach to improve Cd resistance in clove basil.

More upregulated DEGs were enriched in “plant-pathogen interaction” and “MAPK signaling pathway” pathways in the KEGG (Figure 5D). The GO analysis showed that DEGs participated in many significant GO terms in response to Cd stress (Supplementary Figure S7). Similar results were observed in *Paspalum vaginatum* Swartz leaves subjected to Cd stress in a former study (Xu et al., 2022). These results suggest that clove basil made systematic and coordinated responses to adapt to Cd stress. Therefore, the functional annotation of Cd-responsive genes could provide key genes for genetically reforming clove basil with strong Cd resistance, which will facilitate the bioremediation and effective utilization of Cd-contaminated lands.

### Major TFs and genes induced by Cd treatment and their possible roles in regulating Cd tolerance in clove basil

Transcriptomic analyses in various plant have revealed the involvement of many TFs in the transcriptional regulation of Cd-responsive genes (Zhu et al., 2018; Fasani et al., 2019; Wang et al., 2021b). In this study, identified Cd-responsive TF genes belonged to different families, including bHLH, WRKY, AP2/ERF, MYB, ZIP, HSF, and GRAS (Figure 6A), indicating the complexity of plant responses to Cd stress. Most of these TF genes were repressed by Cd stress in clove basil leaves, demonstrating the inhibitory effects of Cd stress on plants at the transcriptional regulation level. This result aligns with the morpho-physiological data, demonstrating that Cd had a detrimental effect on the growth of clove basil. Another explanation for this result is that the downregulated TF genes might have negative regulatory roles in gene expression during Cd stress adaptation.

In tea plants, transcriptomic analysis showed that many DEGs were produced after 10 or 15 days of Cd exposure, including one gene encoding an ERF protein that was positively correlated with five structural genes, including three *CsGolS* family genes, one *CsNCED*, and one *CsHIPP*, using weighted gene co-expression network analysis (Liu et al., 2023). In *Arabidopsis thaliana*, *AtMYB59* was induced by Cd treatment, and the genes directly and indirectly regulated by AtMYB59 were mainly involved in calcium homeostasis and signaling through transcriptomic analysis (Fasani et al., 2019). In soybean plants, 26 Cd-responsive *WRKY* genes were upregulated, while 3 were downregulated by Cd treatment. Among these, *WRKY142* could activate the expression of three Cd tolerance genes, *ATCDT1*, *CDT1-1*, and *CDT1-2*, by directly binding to the W-box element in their promoters (Cai et al., 2020). These reports, together with our results, suggest that Cd-responsive TF genes may play important transcriptional regulatory roles in the expression of HM-stress-related genes.

Recently, several bHLH TFs have been identified as involved in the regulation of Cd tolerance in plants. The ORG3-like gene *GmORG3*, a bHLH family gene, was significantly induced by external Cd stress in soybean plant, and overexpression of *GmORG3* enhanced Cd tolerance and stabilized Fe homeostasis (Xu et al., 2017). Plants overexpressing *bHLH104* exhibited enhanced Cd tolerance in *Arabidopsis thaliana* (Yao et al., 2018). In this study, among 17 Cd-regulated bHLH family genes, 6 *bHLH* genes (c58694.graph_c0, c57229.graph_c0, c56571.graph_c1, c51522.graph_c0, c62303.graph_c0, and c51250.graph_c0) showed significantly increased expression levels after Cd treatment (Figures 6B–G). A novel gene encoding a MYB TF was identified in rice seedlings exposed to Cd stress through comparative transcriptomic analysis, which might be a candidate target for generating Cd-resistant plants (Chen et al., 2021). These results suggested that upregulated *bHLH* TF genes might play crucial regulatory roles in the adaptation of clove basil to Cd stress. These genes could potentially serve as candidate TF genes for developing Cd-resistant clove basil.

The *Lamiaceae* family encompasses a wide variety of plants with biomedical and industrial applications, offering significant potential for medical and daily industries (Uritu et al., 2018). Yet, only limited genetic engineering attempts have been reported in plants belonging to the *Lamiaceae* family. Overexpressing *SmNAC1* enhanced tolerance to high concentrations of Zn ions, and Zn ions were significantly enriched in shoot tissues of *Salvia miltiorrhiza*, a member of the *Lamiaceae* family (Zhu et al., 2019). Treatment with copper sulfate sharply induced the expression of three *SOD* genes, and the authors suggested that these induced *SOD* genes were the main contributors during Cu stress (Han et al., 2020). Furthermore, it has been reported that some members of bHLH, WRKY, and MYB TF families in plants of the *Lamiaceae* family participated in the Cd tolerance by regulating the activities of enzymes, such as PCS and antioxidant enzymes (Raza et al., 2020; Fan et al., 2023). Introducing *Perilla frutescens* genes into tobacco developed a new variety *N. tabacum* L. var. ZSY, which significantly enhanced Cd tolerance, reflecting higher SOD and CAT activities and more alkaloids in plant tissues (Wei and Guo, 2023). In a previous study, we investigated transcriptomic changes in peppermint young plants when exposed to different intensities of Cd stresses and identified several key genes, such as *bHLH47* and *bHLH100*, that were significantly induced by Cd stress and had potential roles in Cd resistance acquisition (Wang et al., 2023). However, their regulatory roles in Cd resistance have not been thoroughly investigated. Therefore, there is a necessity for extensive studies on molecular investigations and genetic improvements for plants in the *Lamiaceae* family to confer HM tolerance.

### Cd treatment affects the antioxidant activity and induces GSH synthesis in clove basil

The activity of antioxidant enzymes is closely related to Cd tolerance in plants. However, the effects of Cd stress on the activity of antioxidant enzymes differ with plant species, tissues, Cd concentration as well as stress duration. For example, Cd toxicity tends to minimize the activities of SOD and CAT in pea (Sandalio et al., 2001) and SOD activity in *Pistia stratiotes* (Zhao et al., 2023). Cd stress at 14 mg/L significantly enhances the activities of POD, SOD and CAT in both leaves and roots of *Dendrobium officinale* seedlings (Jiang et al., 2020), while Cd stress resulted in a significant decline in the activities of GPX, ascorbate peroxidase and CAT in radish (El-Beltagi et al., 2010). In a previous study, we found that long-term exposure to low Cd concentrations induced antioxidant activity in peppermint leaves, while high-intensity Cd stress inhibited that after long-term exposure (Wang et al., 2023).

In this study, Cd stress did not affect SOD activities, reduced TSP contents and POD activities, but enhanced CAT activities in clove basil (Supplementary Figure S8). These results imply that CAT might play a dominant role in scavenging ROS in clove basil during Cd stress adaptation as only CAT activity was enhanced following Cd treatment. Merely having high CAT activity is insufficient to counteract the Cd-induced ROS burst in clove basil. As a result, seedlings continued to exhibit noticeable harmful effects under Cd stress. The expression patterns of genes encoding these enzymes differed among specific genes, suggesting that Cd stress affected compositions in the antioxidant system at both protein and gene levels in clove basil, and they were complementary to each other in coding for proteins.

GSH is a derivative of glutamic acid, cysteine, and glycine amino acids, which has been considered a ligand that chelates HMs in plants (Al-Khayri et al., 2023). Increased GSH synthesis has been reported in *Solanum nigrum* (Deng et al., 2010), *Bacopa monnieri* (Singh et al., 2006), and *Lepidium sativum* (Gill et al., 2012), with increasing Cd concentrations along with enhanced antioxidant activity. In this study, although we did not measure GSH contents in clove basil after Cd treatment, the expressions of two genes involved in the GSH biosynthesis pathway were significantly upregulated by Cd treatment, while the expressions of genes involved in the process of GSH oxidation were repressed (Supplementary Figure S9). Therefore, we can reasonably assume that Cd stress induced GSH biosynthesis but inhibited GSH oxidation in clove basil leaves, resulting in the accumulation of reduced GSH substances that improved antioxidant ability to alleviate Cd-induced oxidative stress and provide sufficient GSH for the generation of phytochelatins (PCs).

Cd stress can induce the generation of PCs, which bind Cd and form varied complexes to reduce Cd toxicity in plant cells. The Cys thiolic groups of PCs guard the cytosol from free Cd ions and eventually sequester Cd in the vacuole (Al-Khayri et al., 2023). In this study, we found that Cd stress significantly induced the expression of two *PCS* genes. Additionally, many *GST* genes showed significant expression after Cd treatment (Supplementary Figure S9). These results together suggest that Cd treatment promoted the biosynthesis of PCs and indicated the important roles of PCs in reducing the effects of Cd toxicity in clove basil.

**FIGURE 7:**
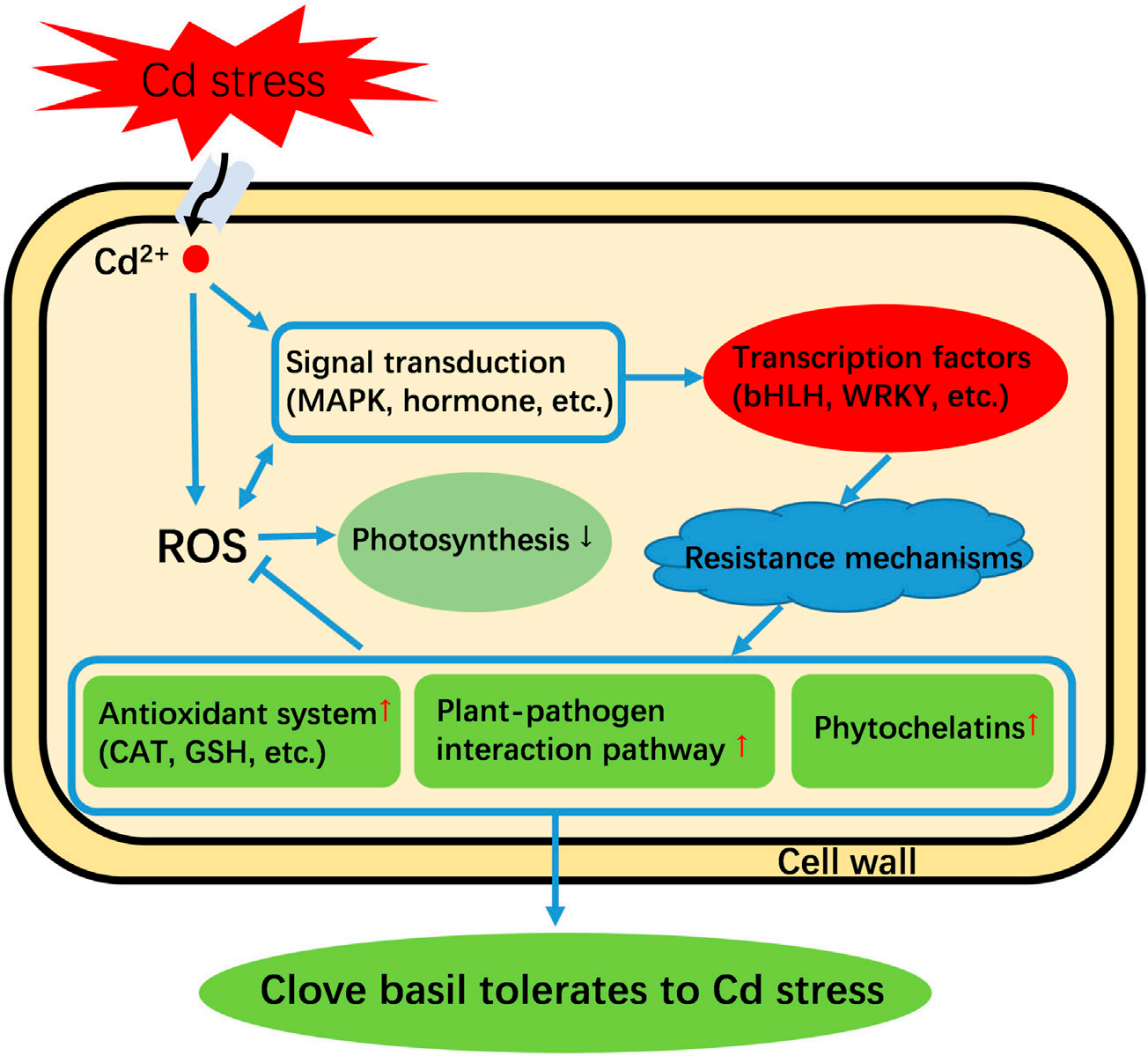
An assumed model of Cd tolerance in clove basil. “↑” means this pathway is strengthened. “↓” means this pathway is repressed. “→” means an induction effect. “丅” means an inhibitory effect.

Based on the above discussions and expression profiles of genes in response to Cd treatment, we developed a hypothetical model of Cd tolerance in clove basil under Cd stress (Figure 7). When seedlings were subjected to Cd stress, it activated signaling transduction pathways and induced ROS bursts in the cell. The increased levels of ROS cause severe oxidative damage to the photosynthetic system in leaves of clove basil seedlings. The activated signals subsequently regulate the expression of key TF genes, such as *bHLH*, to regulate the expression of genes in stress defense pathways. Finally, the activated defense system enables clove basil seedlings to endure Cd stress.

## Conclusion

Long-term Cd stress caused damage to the photosynthetic and root systems of clove basil, resulting in an insufficient energy supply and reduced performance. A large number of Cd-responsive genes in clove basil were identified, providing valuable resources for genetic and genomic studies of clove basil. More importantly, the results of this study suggest that *bHLH* family TF genes might play crucial roles in the adaptation regulations under Cd stress. Therefore, additional investigations are needed to study the functional roles of these Cd-stress inducible genes, especially TF genes, in the regulation of Cd tolerance in clove basil through transgenic methods in the future. Cd treatment induced CAT activity at both protein and gene levels, suggesting that Cd treatment enhances H_2_O_2_ scavenging activity in clove basil leaves because CAT is a key antioxidant enzyme that scavenges H_2_O_2_, and Cd’s toxic effects on plants mainly result from trigged ROS bursts by Cd stress. Overall, our study provides new genetic resources for breeding new cultivars with higher Cd resistance, which would greatly facilitate phytoremediation and effective utilization of Cd-polluted soils.

## Data Availability

The datasets presented in the study are deposited in the NCBI Sequence Read Archive (SRA) repository (https://www.ncbi.nlm.nih.gov/sra). Accession number PRJNA904532.
