# Supplementary material for: Comparative transcriptomic analysis provides key genetic resources in clove basil (Ocimum gratissimum) under cadmium stress
Source: Front Genet. 2023 Jul 27;14:1224140. doi: 10.3389/fgene.2023.1224140 (PMC10412823; doi:10.3389/fgene.2023.1224140)
Supplement: Supplementary file 1 [file Table4.DOC]

Supplementary Table 4. Annotated results of unigenes by blasting several public databases.

| Databases | Annotated Number | 300<=length<1000 | length>=1000 |
| --- | --- | --- | --- |
| COG | 9606 | 2703 | 5395 |
| GO | 33653 | 11607 | 14274 |
| KEGG | 26168 | 8557 | 12005 |
| KOG | 21510 | 6891 | 9885 |
| Pfam | 26052 | 8169 | 13479 |
| Swissprot | 26681 | 8889 | 12403 |
| TrEMBL | 39794 | 14047 | 16646 |
| eggNOG | 33797 | 11675 | 14801 |
| nr | 40191 | 14083 | 16642 |
| All annotated | 41060 | 14425 | 16718 |

Note: Cd concentration in the Control was 0 mg/L.
