# Supplementary figures and images for: Comparative transcriptomic analysis provides key genetic resources in clove basil (Ocimum gratissimum) under cadmium stress
Source: Front Genet. 2023 Jul 27;14:1224140. doi: 10.3389/fgene.2023.1224140 (PMC10412823; doi:10.3389/fgene.2023.1224140)

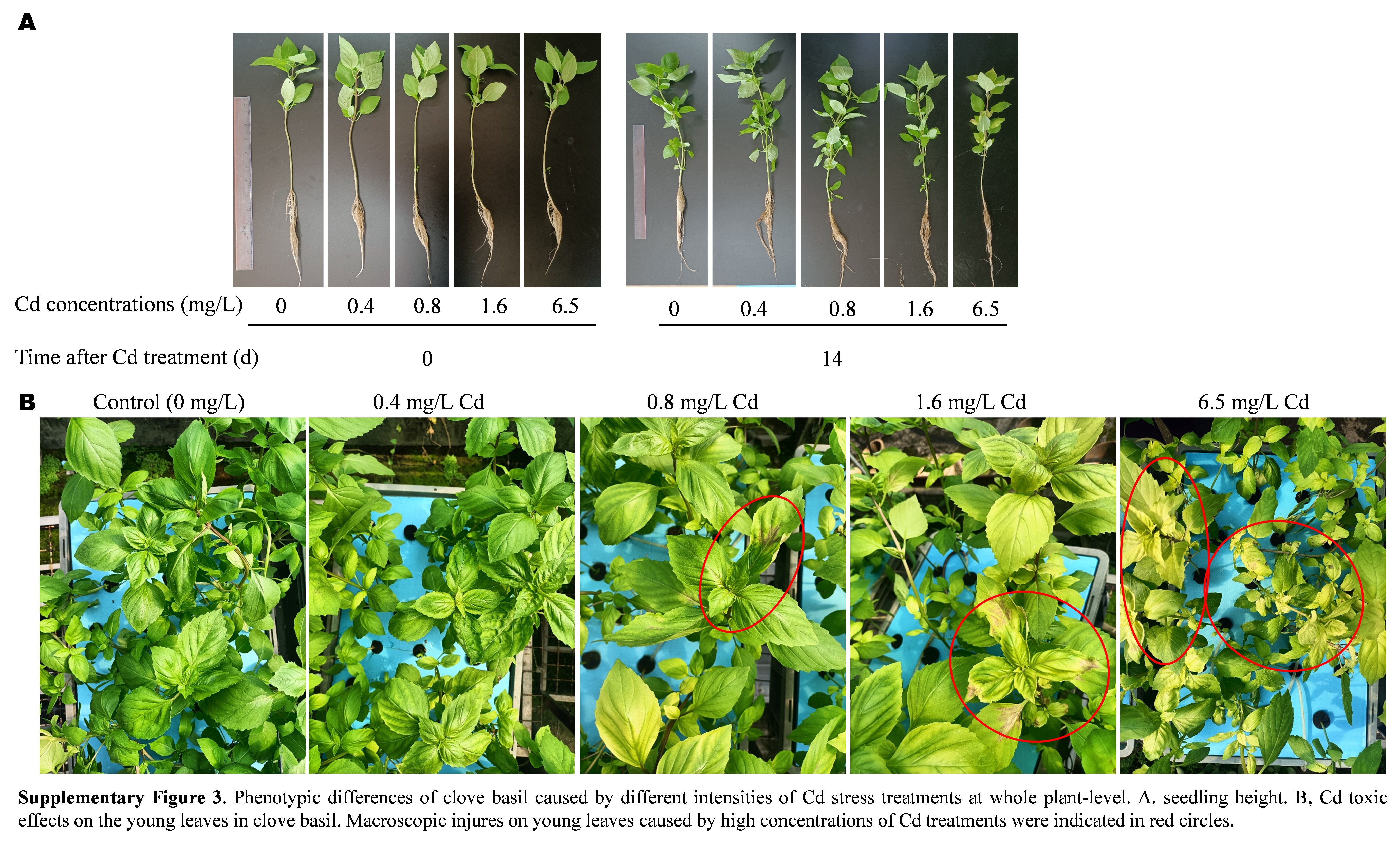

Supplement: Supplementary file 2 [file Image3.JPEG]

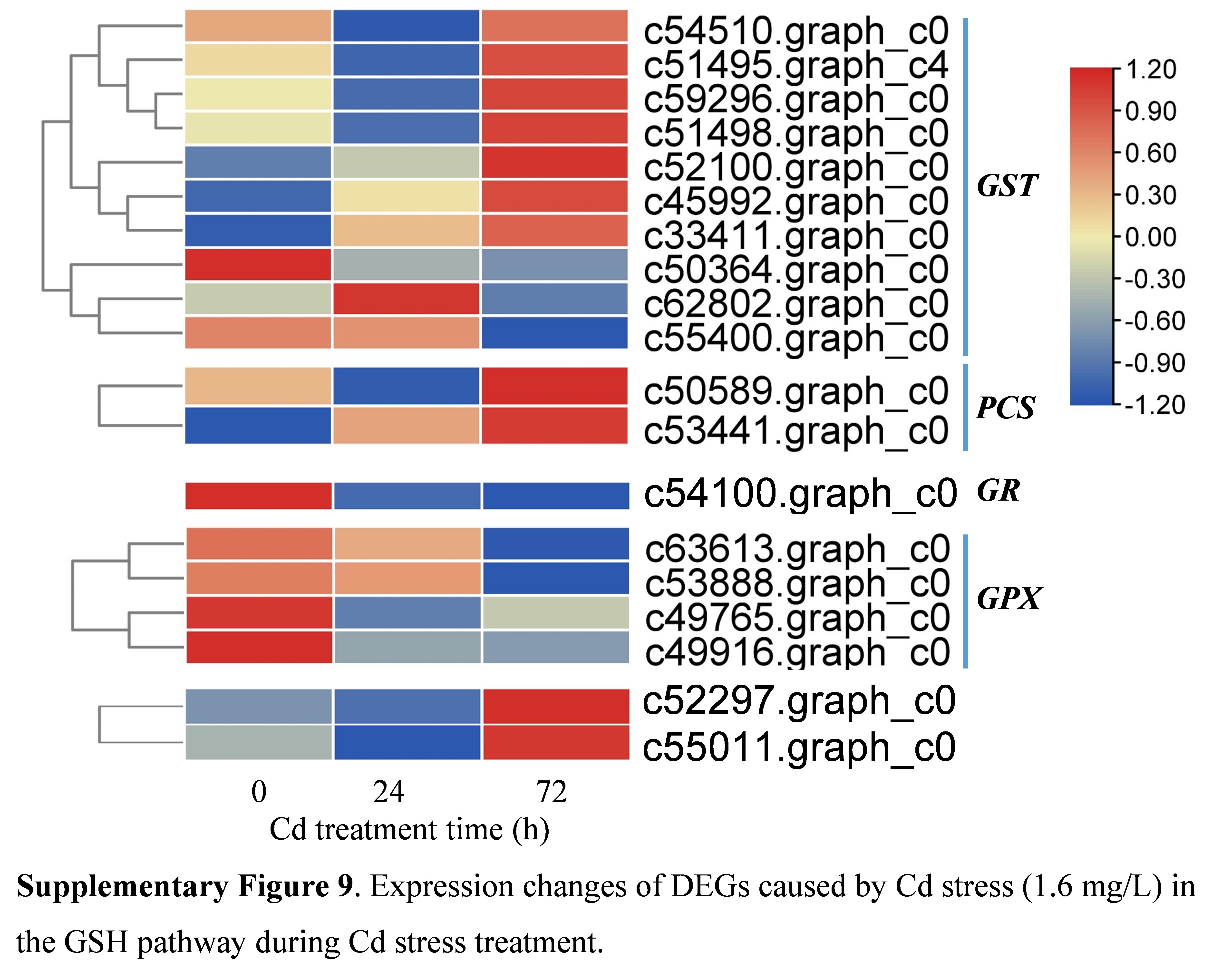

Supplement: Supplementary file 3 [file Image9.JPEG]

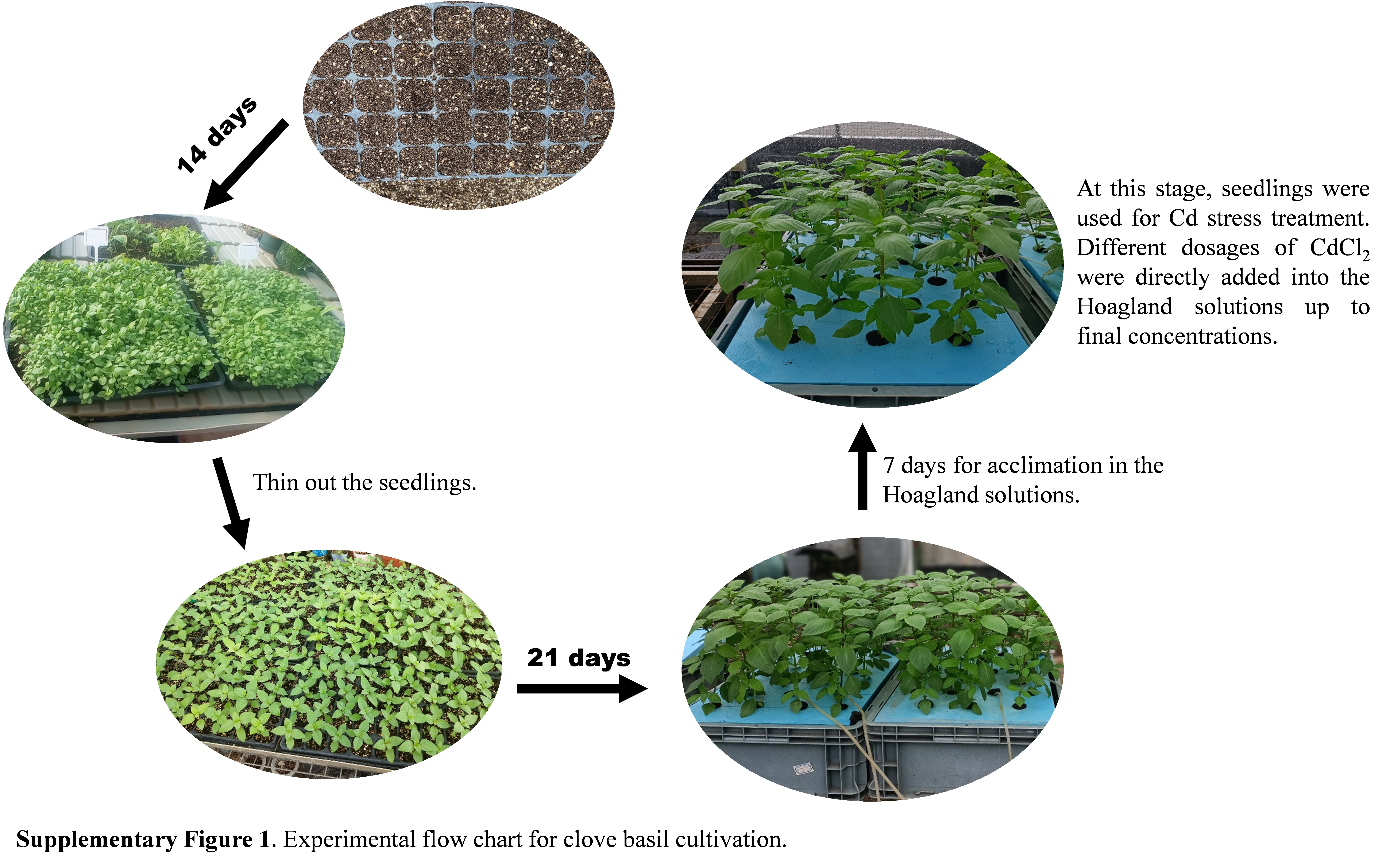

Supplement: Supplementary file 5 [file Image1.JPEG]

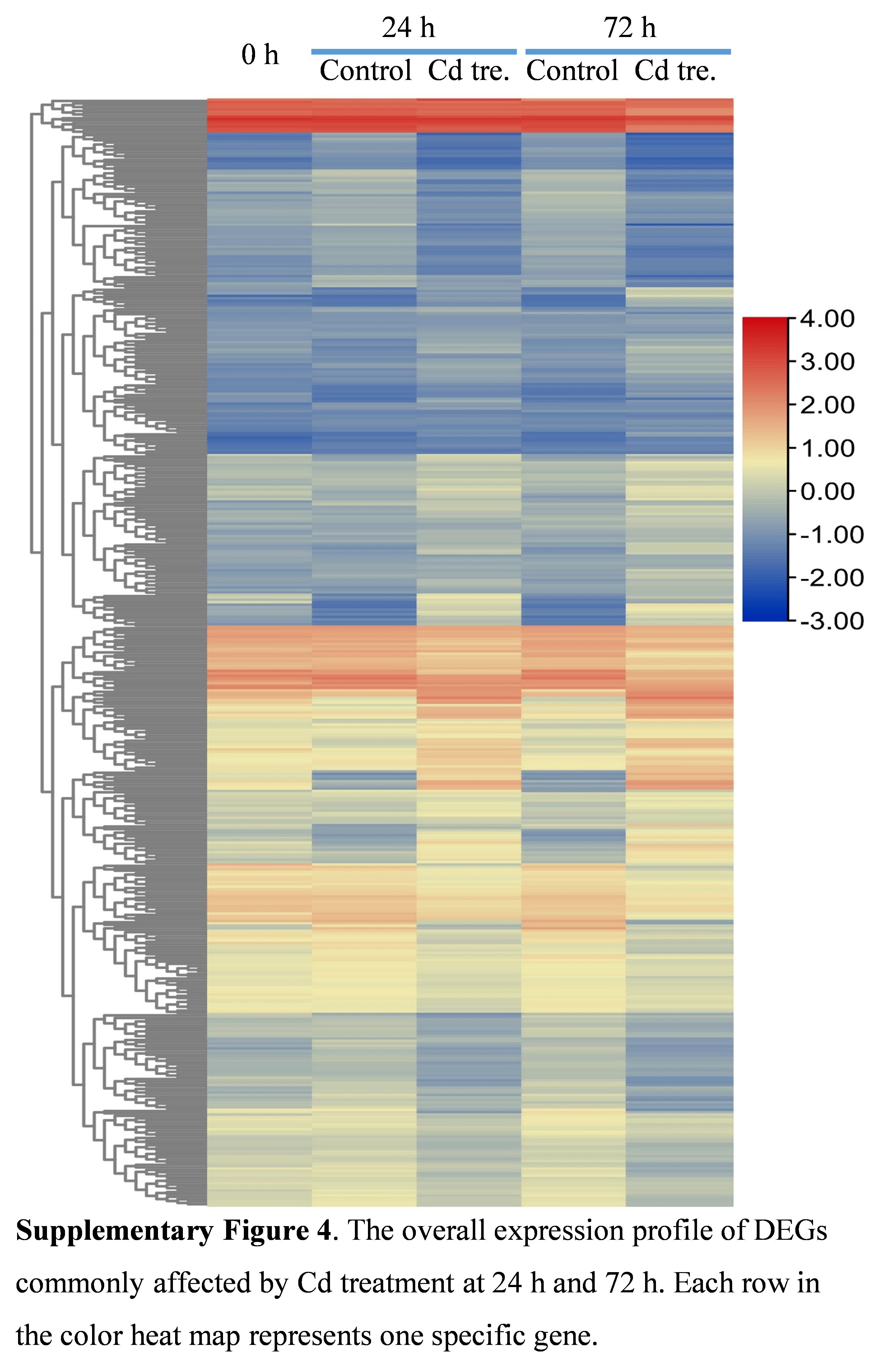

Supplement: Supplementary file 6 [file Image4.JPEG]

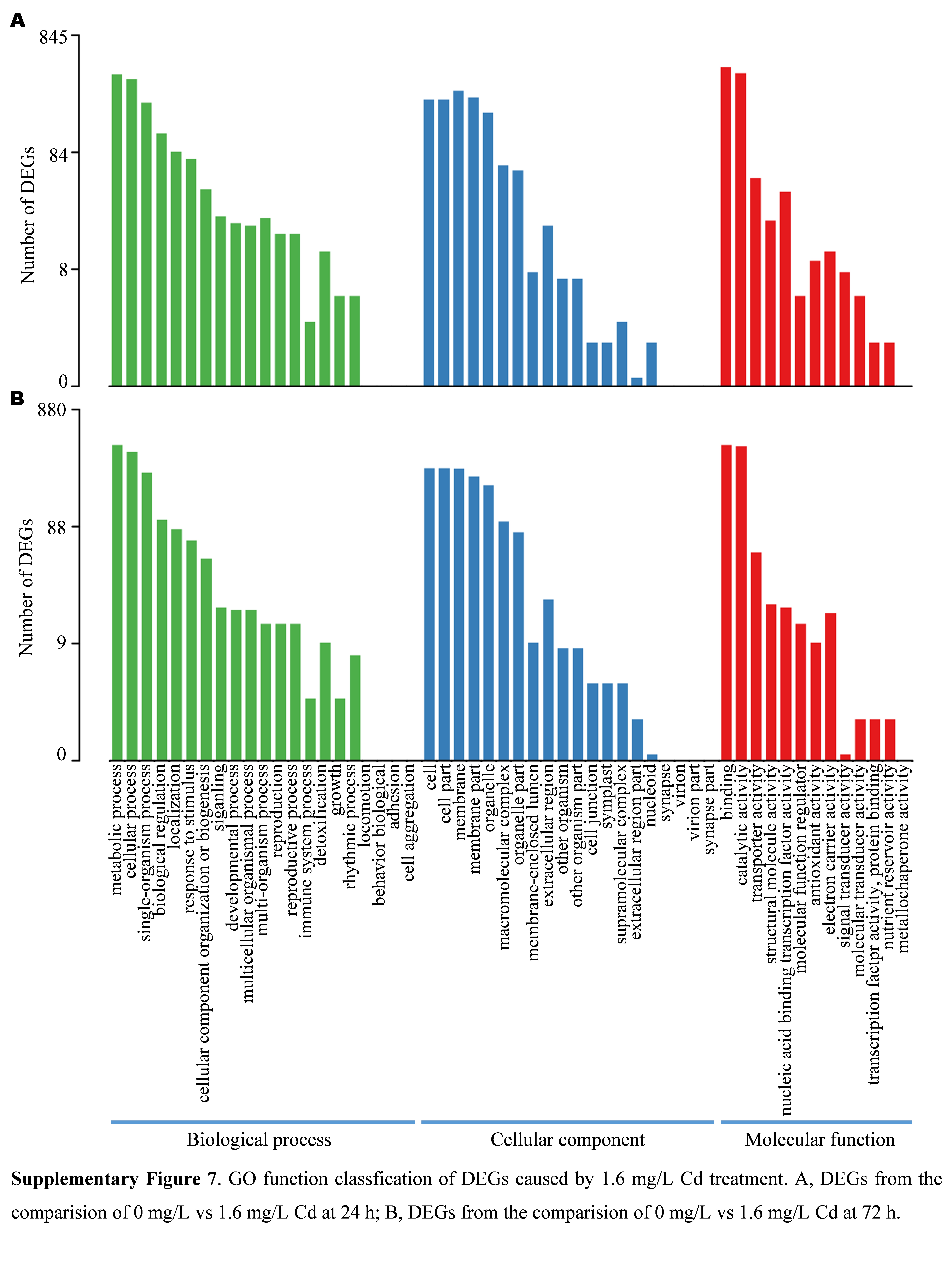

Supplement: Supplementary file 7 [file Image7.JPEG]

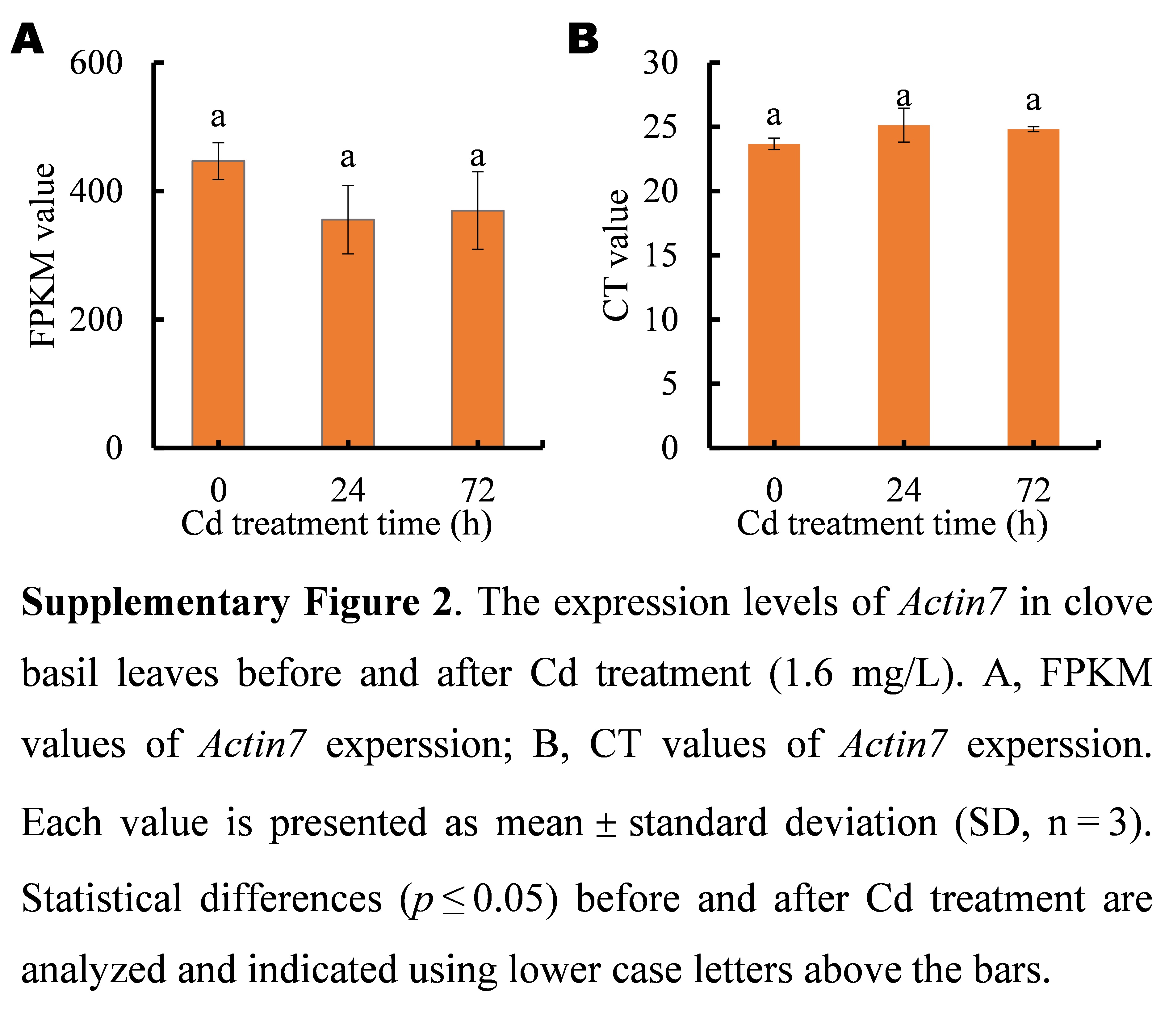

Supplement: Supplementary file 8 [file Image2.JPEG]

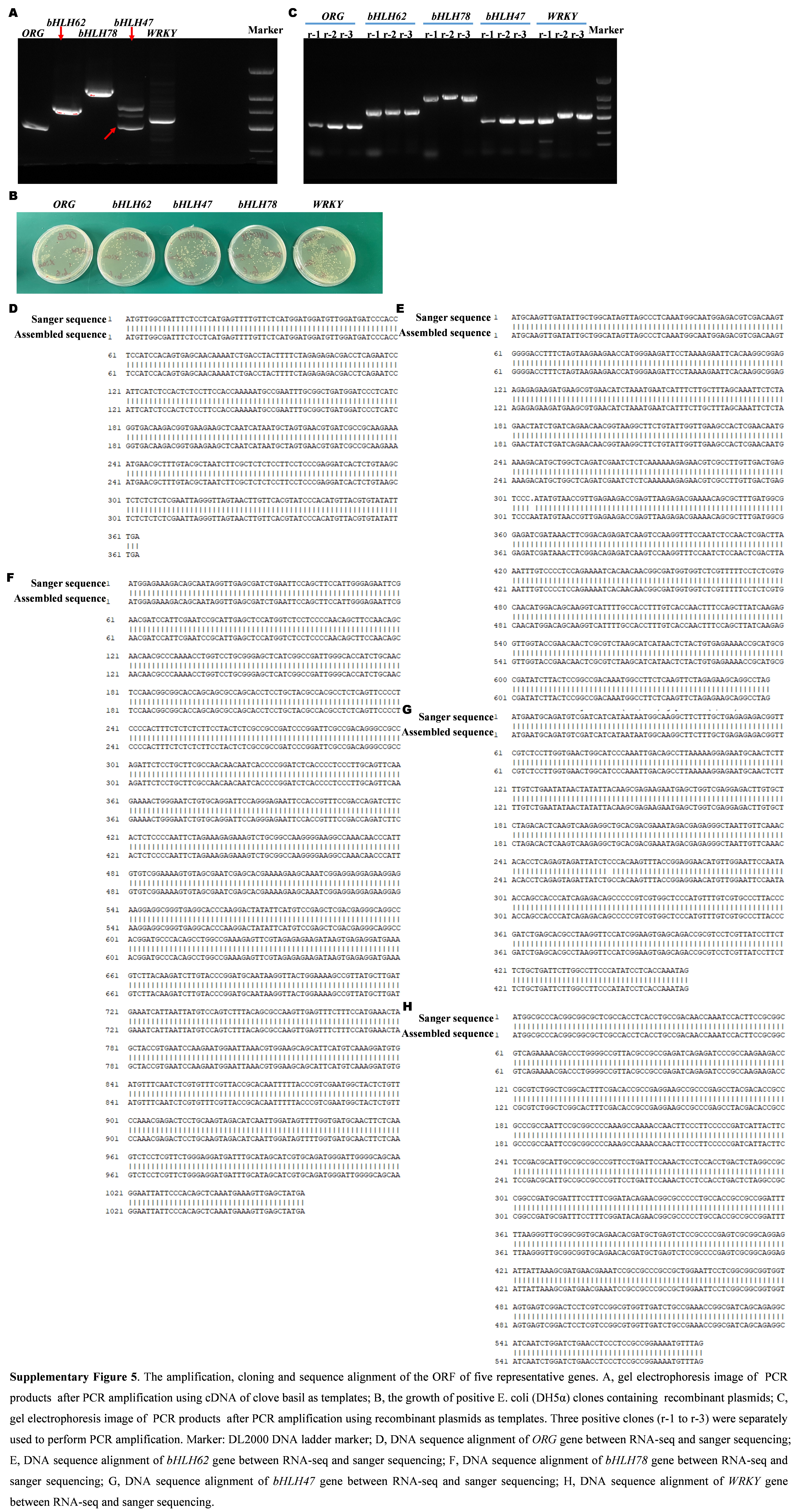

Supplement: Supplementary file 9 [file Image5.JPEG]

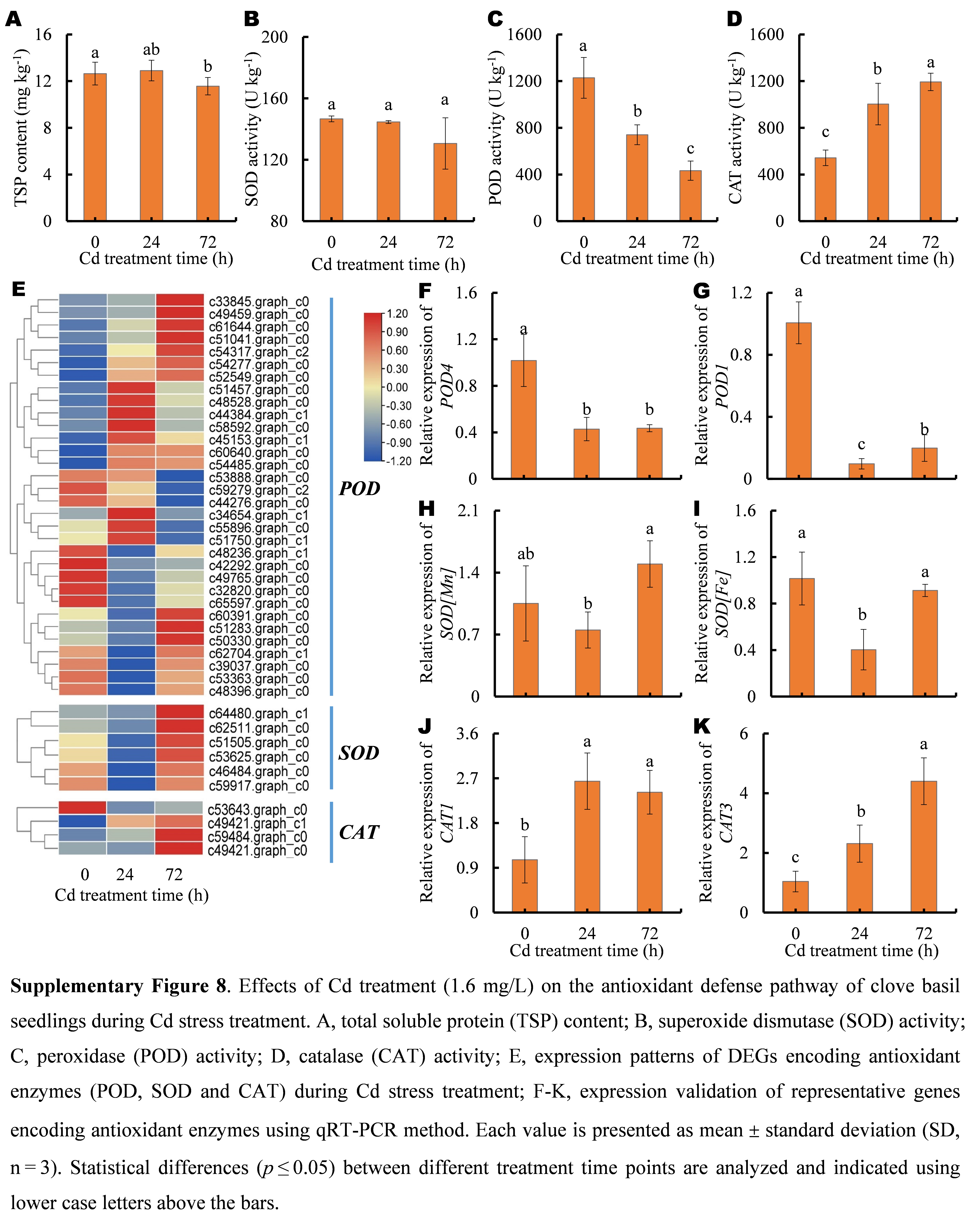

Supplement: Supplementary file 14 [file Image8.JPEG]

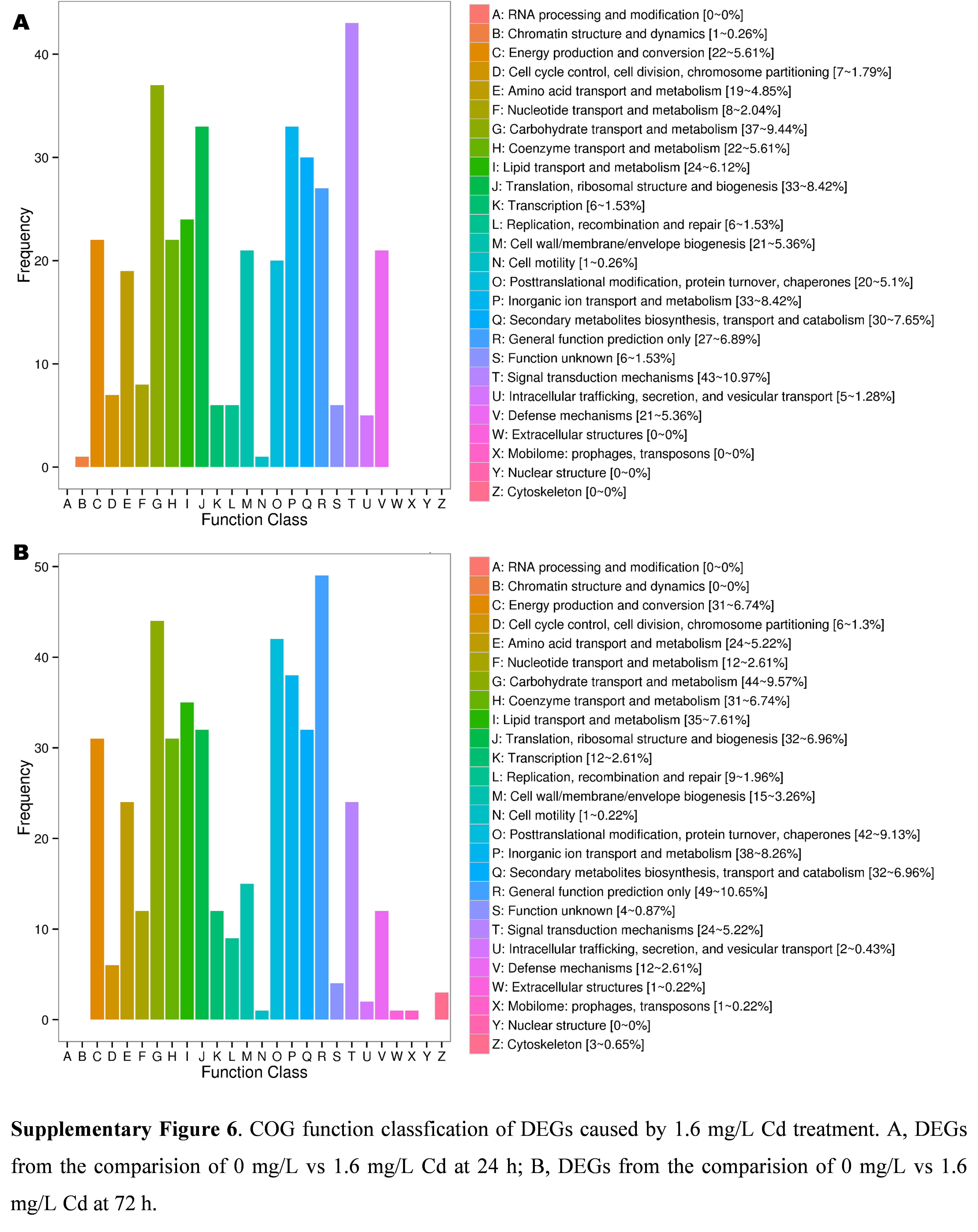

Supplement: Supplementary file 15 [file Image6.JPEG]
