# Supplementary material for: Comparative transcriptomic analysis provides key genetic resources in clove basil (Ocimum gratissimum) under cadmium stress
Source: Front Genet. 2023 Jul 27;14:1224140. doi: 10.3389/fgene.2023.1224140 (PMC10412823; doi:10.3389/fgene.2023.1224140)
Supplement: Supplementary file 4 [file Table3.DOC]

Supplementary Table 3. Statistics of assembly.

| Length Range (bp) | Transcript | Unigene |
| --- | --- | --- |
| 200-300 | 37,960(11.76%) | 27,717(34.81%) |
| 300-500 | 36,700(11.37%) | 18,676(23.45%) |
| 500-1000 | 60,325(18.69%) | 14,423(18.11%) |
| 1000-2000 | 98,732(30.58%) | 9,776(12.28%) |
| 2000+ | 89,124(27.61%) | 9,043(11.36%) |
| Total Number | 322,841 | 79,635 |
| Total Length | 481,771,788 | 65,933,288 |
| N50 Length | 2,174 | 1,650 |
| Mean Length | 1492.29 | 827.94 |

Note: Cd concentration in the Control was 0 mg/L.
