# Supplementary material for: Comparative transcriptomic analysis provides key genetic resources in clove basil (Ocimum gratissimum) under cadmium stress
Source: Front Genet. 2023 Jul 27;14:1224140. doi: 10.3389/fgene.2023.1224140 (PMC10412823; doi:10.3389/fgene.2023.1224140)
Supplement: Supplementary file 10 [file Table1.DOC]

Supplementary Table 1. Primer information for qRT-PCR analysis in this study.

| Usage | Genes | #ID in transcriptomic data | Forward primer sequence (5'-3') | Reverse primer sequence (5'-3') | Product size(bp) |
| --- | --- | --- | --- | --- | --- |
| qRT-PCR | *OT3* | c64691.graph_c0 | TGCCTCAATGGTGGTTCCTC | GAAGCTGCACGTCGTCTTTC | 90 |
|  | *ZT10* | c63312.graph_c0 | ACCGGATTCATGCACGTCTT | GAGGGTGATGATGGCGGAAA | 126 |
|  | *WAT1* | c54509.graph_c1 | AATGTGTGGCTGCTGGGATT | TCTGTAGCGTATCGTGTGGC | 91 |
|  | *AAT* | c54570.graph_c0 | GGGGTCGGAACAGACCAAAT | CATAACCGCTACTCCGTCCC | 75 |
|  | *Hsp90* | c54531.graph_c1 | GGCCGTCGAAGAGATCGAAT | AAGCACTTCTCCGTTGAGGG | 85 |
|  | *Hsp20* | c47329.graph_c0 | GGTCCAATCCCATCACCCTG | CCACACATCCACCCACAACT | 104 |
|  | *GST3* | c50364.graph_c0 | GCAGCTTGAGGAGAAACCCT | AAAACGGAATCAACGCCACG | 71 |
|  | *GST1* | c59296.graph_c0 | AATCATTGCTGCCCATCCCA | TGAATGGCGGTGCTCAAGAT | 75 |
|  | *SOD[Cu-Zn]* | c62511.graph_c0 | CTCGGAAAAGGTGGTCACGA | CTCACAGGGGAGTCAAACCC | 91 |
|  | *Actin7* | c52716.graph_c0 | GGAGCTCGTCTTTGCTGTCT | GAGCGGGAAATTGTGAGGGA | 90 |
|  | *POD4* | c32820.graph_c0 | CGGAAGTTCCATTCCACCCA | CTTAGGTGGTCCGAACCCTG | 94 |
|  | *POD1* | c49765.graph_c0 | AACAAGAGGGCGGAAGGAAG | AGAGAAAGAGGGGCTGTTGC | 73 |
|  | *SOD[Mn]* | c46484.graph_c0 | TTAGGGTTCGAAGCGCCATT | CACGTGTGTGAGACCGAGAA | 127 |
|  | *SOD[Fe]* | c59917.graph_c0 | CCCACATCGGGGTGAAAGAA | GCTCGAGTTCGTCCGTACAT | 72 |
|  | *CAT1* | c49421.graph_c1 | CCAGATGCTGCGGATCTCAT | TTGATCCAGCCAGGCAAGAG | 89 |
|  | *CAT3* | c59484.graph_c0 | ACCGGTATGTTGTTCCCGAC | CGCCGTTGATCATCCGTTTC | 136 |
| Gene cloning | *ORG* | c58694.graph_c0 | ATGTTGGCGATTTCTCCTCA | TCAAATATACACGTAACATGTGGGA | 363 |
|  | *bHLH62* | c57229.graph_c0 | ATGCAAGTTGATATTGCTGGC | CTAGGCCTGCTTCTCTAGAACT | 657 |
|  | *bHLH78* | c56571.graph_c1 | ATGGAGAAAGACAGCAATAGGTTGA | TCATAGCTCAACTTTCATTTGAGCTG | 1059 |
|  | *bHLH47* | c51522.graph_c0 | ATGAATGCAGATGTCGATCATCA | CTATTTGGTGAGGATATGGGAAGG | 459 |
|  | *WRKY* | c63519.graph_c0 | ATGGCGCCCACGGCGGCG | CTAAACATTTTCCGGCGGAG | 582 |
