# Supplementary material for: Comparative transcriptomic analysis provides key genetic resources in clove basil (Ocimum gratissimum) under cadmium stress
Source: Front Genet. 2023 Jul 27;14:1224140. doi: 10.3389/fgene.2023.1224140 (PMC10412823; doi:10.3389/fgene.2023.1224140)
Supplement: Supplementary file 11 [file Table5.DOC]

Supplementary Table 5. Alignment results of each sample.

| Treatment time (h) | Samples | Clean Reads | Mapped Reads | Mapped Ratio (%) |
| --- | --- | --- | --- | --- |
| 0 | Control-1 | 22,830,057 | 17,200,093 | 75.34 |
|  | Control-2 | 22,391,222 | 17,070,648 | 76.24 |
|  | Control-3 | 21,181,042 | 16,240,183 | 76.67 |
| 24 | Control-1 | 21,485,915 | 16,070,383 | 74.79 |
|  | Control-2 | 21,898,153 | 16,540,511 | 75.53 |
|  | Control-3 | 21,872,539 | 16,650,456 | 76.12 |
|  | 1.6 mg/L Cd-1 | 19,879,725 | 15,360,130 | 77.27 |
|  | 1.6 mg/L Cd-2 | 26,515,610 | 20,152,570 | 76.00 |
|  | 1.6 mg/L Cd-3 | 22,180,513 | 17,217,003 | 77.62 |
| 72 | Control-1 | 22,127,839 | 16,819,392 | 76.01 |
|  | Control-2 | 20,937,711 | 15,897,564 | 75.93 |
|  | Control-3 | 19,962,929 | 15,148,810 | 75.88 |
|  | 1.6 mg/L Cd-1 | 26,273,891 | 19,879,591 | 75.66 |
|  | 1.6 mg/L Cd-2 | 22,484,498 | 16,981,020 | 75.52 |
|  | 1.6 mg/L Cd-3 | 20,198,022 | 15,301,239 | 75.76 |

Note: Cd concentration in the Control was 0 mg/L.
