# Supplementary material for: Comparative transcriptomic analysis provides key genetic resources in clove basil (Ocimum gratissimum) under cadmium stress
Source: Front Genet. 2023 Jul 27;14:1224140. doi: 10.3389/fgene.2023.1224140 (PMC10412823; doi:10.3389/fgene.2023.1224140)
Supplement: Supplementary file 13 [file Table2.DOCX]

Supplementary Table 2. Sequencing reads statistics after filtering.

| Treatment time (h) | Samples | Read Number^@^ | Base Number^#^ | GC Content (%) | ≥Q30 (%)^*^ |
| --- | --- | --- | --- | --- | --- |
| 0 | Control-1 | 22,830,057 | 6,835,741,956 | 49.84 | 93.02 |
|  | Control-2 | 22,391,222 | 6,701,071,136 | 49.80 | 92.93 |
|  | Control-3 | 21,181,042 | 6,333,596,332 | 50.05 | 93.88 |
| 24 | Control-1 | 21,485,915 | 6,430,661,046 | 50.28 | 93.29 |
|  | Control-2 | 21,898,153 | 6,544,396,258 | 50.27 | 93.37 |
|  | Control-3 | 21,872,539 | 6,543,576,864 | 50.81 | 93.37 |
|  | 1.6 mg/L Cd-1 | 19,879,725 | 5,946,242,484 | 49.89 | 93.52 |
|  | 1.6 mg/L Cd -2 | 26,515,610 | 7,939,038,498 | 49.85 | 93.39 |
|  | 1.6 mg/L Cd -3 | 22,180,513 | 6,634,646,574 | 49.90 | 93.62 |
| 72 | Control-1 | 22,127,839 | 6,621,025,914 | 49.96 | 93.08 |
|  | Control-2 | 20,937,711 | 6,263,190,330 | 50.20 | 93.83 |
|  | Control-3 | 19,962,929 | 5,968,010,970 | 50.29 | 93.46 |
|  | 1.6 mg/L Cd-1 | 26,273,891 | 7,865,921,588 | 48.72 | 93.00 |
|  | 1.6 mg/L Cd -2 | 22,484,498 | 6,725,488,790 | 48.87 | 92.64 |
|  | 1.6 mg/L Cd -3 | 20,198,022 | 6,045,738,408 | 48.78 | 93.47 |

^@^the number of paired-end reads in the clean data.

^#^ total base number of clean data.

^*^ base which quality value is greater than or equal to 30 percentage of total clean data.

Note: Cd concentration in the Control was 0 mg/L.
